# Linking magnetic susceptibility with polycyclic aromatic hydrocarbon concentrations in urban road dust: a proxy-based approach for contamination monitoring

**DOI:** 10.1007/s10653-026-03240-6

**Published:** 2026-05-11

**Authors:** Sylwia Dytłow, Jakub Karasiński

**Affiliations:** 1 Institute of Geophysics, Polish Academy of Sciences, Ks. Janusza 64, 01-452 Warsaw, Poland; 2 Faculty of Chemistry, Biological and Chemical, Research Centre, University of Warsaw, Warsaw, Poland

**Keywords:** Road dust, Magnetic susceptibility, PAH, PCA, Urban contamination, Ecological risk

## Abstract

This study evaluated 57 road dust samples collected in Warsaw, Poland, focusing solely on the < 0.2 mm grain size fraction for the analysis of polycyclic aromatic hydrocarbons (PAHs). The total PAH concentration (∑16PAH) ranged from < LOQ to 12.0 mg/kg (mean = 3.56 mg/kg), with the highest levels observed in areas with dense traffic and limited dispersion of contamination. Acenaphthene (Ace), fluorene (Flu), and phenanthrene (Phe) were the most abundant compounds, while high-molecular-weight PAHs constituted approximately 55.2% of the total PAHs. Diagnostic isomer ratios (e.g., IP/(IP + BghiP)) consistently indicated mainly pyrogenic origins. Magnetic susceptibility (χ), normalized for the proportion of fine particles (χ_WN_), was strongly correlated with both total PAHs (r = 0.792, R^2^ = 0.628) and high-molecular-weight PAHs (r = 0.797, R^2^ = 0.635), confirming that χ_WN_ can serve as a robust proxy as a robust proxy for PAH contamination in road dust. Multivariate analyses revealed distinct contamination patterns and grouped PAHs based on their shared sources and physicochemical properties. An ecological risk assessment (MERM-Q) revealed that most samples fell into the low-risk category. The highest risk values were recorded in the city center, where the traffic intensity exceeded 35,000 vehicles/day. These findings demonstrate the potential of χ_WN_ as a low-cost, non-destructive screening tool to support more efficient environmental monitoring and optimize the selection of sampling sites for detailed PAH analysis.

**Supplementary Information:**

The online version contains supplementary material available at 10.1007/s10653-026-03240-6.

## Introduction

Polycyclic aromatic hydrocarbons (PAHs) are a class of hazardous contaminants that have raised global environmental and health concerns due to their persistence, toxicity, and widespread distribution in urban environments (Cachada et al., 2016; Gan et al., 2009). In urban settings, PAH are commonly associated with road surfaces, where they adsorb onto road-deposited particles, collectively referred to as road dust. Road dust is a major reservoir of PAHs, which are subsequently transported into water bodies via surface runoff, leading to declines in water quality (Amato et al., 2011; Liu et al., 2017; Majumdar et al., 2012). PAHs are primarily introduced into the environment through anthropogenic processes such as the incomplete combustion of organic matter, emissions from motor vehicles, and the release of petroleum-derived substances (Anh et al., 2019; Boonyatumanond et al., 2007; Dat et al., 2017, 2024; Gope et al., 2020; Xu et al., 2022; Zhao et al., 2020).

Environmental magnetic analysis has emerged as a valuable tool for contamination assessment because of its non-destructive nature, high sensitivity, methodological simplicity, and cost-effectiveness, making it broadly applicable in environmental studies. Magnetic susceptibility is a widely recognized parameter used for assessing heavy metal contamination in various environmental media, including soil (Ayoubi & Karami, 2019), road dust (Xie et al., 2000), plant leaves (Khamesi et al., 2020), and airborne particulate matter (Gillooly et al., 2019). Its value lies in its ability to detect magnetic particles of anthropogenic origin, often associated with vehicular traffic and industrial activity. While the application of magnetic susceptibility in tracing metal contamination is well established, relatively few studies have investigated whether magnetic susceptibility could also serve as a proxy for organic contaminants, particularly PAHs**.** While earlier research explored this relationship in other media, such as sediments and soils, to the best of our knowledge, this study is the first to specifically test the relationship between PAH concentrations and magnetic susceptibility in road dust. Morris et al. (1994) demonstrated a strong correlation between PAH concentrations and magnetic susceptibility in sediments from Hamilton Harbor, whereas Versteeg et al. (1995) reported that non-destructive magnetic measurements can effectively delineate contamination boundaries and distinguish disturbed from undisturbed sediments. Halsall et al. (2008) demonstrated that combined magnetic susceptibility and PAH measurements can effectively identify and differentiate between indoor and outdoor sources of contamination. Hanesch and Scholger (2002) demonstrated that anthropogenic contamination is the main factor influencing soil magnetic susceptibility in Vienna, Austria, with a notably high correlation between susceptibility and PAH content. Martins et al. (2007) reported a strong correlation, especially between high-molecular-weight PAHs and magnetic susceptibility, in sediment cores from the Santos Estuary, Brazil. While the χ–PAH relationship has been explored in sediments and soils, its utility for rapid assessment of road dust—a critical vector of human exposure in cities—remains untested, especially in Central European urban areas with mixed emission sources.

Traditional chemical methods for determining PAH contamination are accurate but time-consuming and costly. Therefore, there is a growing need for faster, cost-effective screening tools to support spatial assessment of road dust contamination. Magnetic susceptibility measurement offers significant potential due to its speed, non-destructive nature, and established correlation with combustion-derived contaminants. However, its use as a proxy for PAH content in road dust has not been tested thus far, making this study a novel contribution to environmental monitoring approaches.

The main objectives of this study are as follows:

1. To evaluate the relationship between magnetic susceptibility and PAH concentrations in the fine fraction (< 0.2 mm) of urban road dust collected in Warsaw, Poland;
2. To assess the spatial distribution of individual PAHs across the city;
3. To explore the potential of magnetic susceptibility as a screening tool for optimizing sample selection and identifying areas of elevated PAH contamination;
4. To perform source apportionment of PAHs via diagnostic isomer ratios and advanced statistical methods to identify and quantify the main emission sources contributing to contamination; and (5) to apply multivariate statistical techniques, including principal component analysis (PCA), hierarchical cluster analysis (HCA), and self-organizing maps (SOMs, known as Kohonen maps), to uncover underlying patterns, classify sampling sites, and enhance the interpretation of complex datasets related to PAH contamination.

## Materials and methods

### Study area

The sampling operations were conducted in Warsaw (Figure S1 in Supplementary Materials), the capital of Poland, which is centrally located within the Mazovian region along the Vistula River (52° 13′ 48″ N, 21° 00′ 40″ E). The city covers an area of 517.24 km^2^ and lies at an average elevation of 100 m above sea level within the flat Masovian Plain. Warsaw is characterized by a humid continental climate with distinct seasonal variation: winter temperatures range from − 5 to 0 °C, whereas summer averages range between 19 and 24 °C. The annual precipitation typically ranges from 500 to 600 mm, with the majority occurring between May and September (Kaźmierczak & Czarnecka, 2018). Meteorological data indicate that westerly winds are dominant in the region, occurring approximately 16% of the year (approximately 58 days), followed by southeasterly winds at approximately 10% (36 days annually), whereas northerly winds are the least frequent, occurring only approximately 3% of the year (approximately 11 days) (City of Warsaw, 2018; Ratajczak, 2019).

### Sampling campaign for urban road dust

Road dust samples were collected from 57 locations across the city and were selected to represent a range of land use types and traffic intensities. Sampling was carried out between September and November 2023 on dry days and by the official street cleaning schedule provided by Warsaw City Hall (ZOM, 2025). The sampling dates were planned to ensure that dust was collected as far as possible from recent street cleaning events. To minimize the influence of wind variability and long-range transport on the collected data, road dust sampling was carried out from September to early November, a period characterized by the highest frequency of days with wind speeds below 2 m/s (Kossowska-Cezak & Bareja, 1998; City of Warsaw, 2018). At each site, road dust was collected from paved and unpaved surfaces via a clean vacuum cleaner, brush, and dustpan. Each sample consisted of a composite of subsamples, which were geolocated via a handheld GPS device and stored in polyethylene bags for further analysis. After air-drying, the samples were sieved via a laboratory shaker LPzE-2e (MULTISERW-Morek, Poland) equipped with a 0.2 mm sieve. In this study, only the fine fraction with a grain size less than 0.2 mm was selected for further magnetic and chemical analyses.

### Mass contributions of fine sand and silt (diameter < 0.2 mm)

The < 0.2 mm fraction (fine sand and silt) was selected for analysis due to its high mobility during surface runoff and potential for atmospheric transport. This fraction exhibited significant mass variability across samples, ranging from 2.84 to 50.4% (mean = 19.9%, SD = 12.2%; Figure S2). Direct comparisons with other studies remain challenging due to inconsistent size-fraction thresholds; for example, Zhu et al. (2021) reported a higher median proportion (65.3%) for the < 0.25 mm fraction in China, a discrepancy likely driven by different local geological conditions. To facilitate a physically meaningful correlation between magnetic susceptibility and PAH concentrations, three weight-normalized parameters were introduced: weight-normalized magnetic susceptibility (χWN), weight-normalized total PAH concentration (∑PAH_WN_), and weight-normalized high-molecular-weight PAH concentration (∑HMW-PAH_WN_). Each parameter was calculated by multiplying the respective value measured on the < 0.2 mm fraction by the weight percentage of this fraction in the bulk sample. This normalization was necessary because road dust collected in the field contains coarser grain-size fractions (> 0.2 mm) that contribute to the total bulk mass but carry no magnetic or chemical signal as measured here. Without this correction, both χ and PAH concentrations would be implicitly overestimated relative to the true bulk composition, particularly for samples with low fine fraction contributions (as low as 2.84% in this study). Applying the same normalization factor to both variables ensures that χWN and ∑PAH_WN_ are expressed on a common per-gram-of-bulk-sample basis, reflecting the actual co-variation of magnetic and PAH signals across the full dataset. These parameters were used exclusively for the correlation analysis in Sect. "Weight-normalized magnetic susceptibility (χWN) and weight normalized PAH (∑PAHWN)"; all other analyses were based on raw measured values.

### Magnetic susceptibility (χ) measurement

Magnetic susceptibility (χ) quantifies a material’s ability to become magnetized when subjected to an external magnetic field. This property is influenced by factors such as the concentration and mineralogy of magnetic particles, as well as the presence of fine-grained magnetic minerals (Thompson & Oldfield, 1986). In environmental studies, χ serves as a proxy for anthropogenic magnetic particles, which are typically associated with ferro- and ferrimagnetic iron-bearing compounds originating from contamination sources.

In the present work, samples of street dust were placed in standard plastic containers with a volume of 8 cm^3^ (Vo). Susceptibility measurements were carried out via the multifunctional Kappabridge MFK1-FA system (AGICO, Czech Republic), which operates at a frequency of 976 Hz. The system, characterized by a sensitivity of 2 × 10^−8^ SI and a magnetic field intensity of 200 A/m, allows effective suppression of paramagnetic signals and enhances the detection of ferromagnetic particles, which typically saturate at lower field strengths (Evans & Heller, 2003; Thompson & Oldfield, 1986).

The mass-specific magnetic susceptibility (χ, m^3^ kg^−1^) was calculated via Eq. (1):

1 $$\upchi =\frac{\kappa \times \mathrm{V}\mathrm{o}}{m}$$

where χ is the mass-specific magnetic susceptibility (m^3^ kg^−1^), $\kappa$ is the volume-specific magnetic susceptibility (dimensionless), *V*_*0*_ is the volume of the sample (m^3^), and *m* is the dry mass of the sample (kg).

### Analytical procedure for PAH quantification

#### PAH extraction and analysis

Prior to the quantitative determination of PAHs, the street dust samples were extracted. To ensure data comparability, these analyses were performed using aliquots from the same homogenized samples utilized for magnetic susceptibility measurements Initially, the acetonitrile (ACN) extraction method reported by Puy-Alquiza et al. (2016) was applied; however, this protocol underwent several adjustments. By evaluating the reference material (LGC6188), the impact of extraction duration on recovery was assessed. Furthermore, various preconcentration stages were tested to find an optimal balance between the signal strength and the adverse effects of the matrix, ensuring the most effective signal-to-noise ratio.

In the final procedure, approximately 5 g of the homogenized material was weighed and mixed with 25 mL of ACN. The mixture was subjected to mechanical agitation for approximately 3 min, followed by 2 h of ultrasonic-assisted extraction in a sealed system maintained at 30 °C. To effectively separate the liquid phase from the solid dust particles, the suspension was centrifuged. To effectively separate the liquid phase from the solid dust particles, the suspension was centrifuged at 4200 rpm for 30 min. The resulting supernatant was then evaporated to dryness at 30 °C via a SpeedVac (Eppendorf Concentrator Plus) unit. The choice of 30 °C represented a strategic compromise, providing adequate efficiency while preventing the loss of the most volatile PAHs, even within the closed setup. Finally, the residue was reconstituted with 500 μL of ACN. After a 10 min stabilization period and an additional 2 min of mechanical shaking, the extract was transferred into a chromatographic vial.

#### PAH quantification through chromatographic techniques

PAH concentrations were determined using the chromatographic conditions and detection parameters detailed in Table S1. The total PAH concentration was calculated as the sum of 16 individual compounds (Supelco, CRM47940). Analyses were performed using HPLC (Agilent 1290) coupled with a Diode Array Detector (DAD) and a Triple Quadrupole Mass Spectrometer (Agilent 6460) featuring an APCI source operating in positive ion mode. For the separation, a specialized chromatographic column (Phenomenex, Kinetex 3.5 μm PAH, 100 × 2.1 mm) was used with a gradient elution of 0.100% formic acid in water and 0.100% formic acid in ACN. Detailed parameters for separation and the description of the method optimization process (including wavelength selection and MS/DAD signal comparison) are provided in the Supplementary Material (Section S1. PAH quantification through chromatographic techniques). Following optimization, UV detection at 254 nm with a 400 nm reference was exclusively used for quantification. Identification was based on retention time comparison with the analytical standard, and the protocol was validated using matrix-certified reference materials (LGC6188, NIST2768).

The concentrations of individual PAHs, their cumulative sums, and the respective limits of quantification (LOQ) are given in Table S2 of the Supplementary Materials. Uncertainty associated with the cumulative PAH concentrations was evaluated by error propagation, considering the total as the sum of the 16 individual PAH values. For statistical purposes, values below the LOQ were replaced by LOQ/2, following common practice for left-censored environmental datasets (Antweiler & Taylor, 2008; George et al., 2021). Further technical details regarding the validation of the measurement protocol, quality control frameworks, and the step-by-step uncertainty calculation are provided in the Supplementary Material (Section S2 in Supplementary Materials). The comprehensive mathematical methodology for uncertainty estimation, including the specific error propagation formulas used to calculate the final 14.000% standard uncertainty for the total PAH content, is detailed in the Supplementary Material (Section S3 in Supplementary Materials).

### Principal component analysis (PCA) and hierarchical cluster analysis (HCA)

Principal component analysis (PCA) was used to examine the spatial distribution of PAHs in street dust samples and to identify their possible sources. Furthermore, PCA was applied to analyze the relationships between individual PAH compounds and environmental factors such as magnetic susceptibility and traffic density. This multivariate technique simplifies complex datasets by transforming the original variables into a smaller set of uncorrelated variables, called principal components (PCs), which explain the majority of the variance in the data. HCA was used to group the sampling sites based on the relative content of PAH compounds in the road dust samples. Ward’s method and Euclidean distance were employed to measure the dissimilarity between objects (Ran et al., 2023). The analysis was performed via Statistica 13.3 software.

### Self-organizing map (SOM)-Kohonen map

Self-organizing maps (SOMs) are a type of unsupervised artificial neural network developed by Kohonen (2001) and are commonly used for the visualization and structural analysis of high-dimensional datasets. In this study, SOM was applied to identify patterns and similarities in the distributions of PAHs, traffic intensity, and magnetic susceptibility in road dust samples.

The algorithm projects normalized multidimensional input vectors onto a two-dimensional grid of neurons, preserving the topological relationships between the data points. The SOM analysis was conducted via Statistica 13.3, which has a 5 × 5 neuron structure. The size of the SOM grid (5 × 5 neurons) was selected on the basis of the number of samples and variables to balance the resolution of the mapping and avoid overfitting. A SOM network of size 18 × 25 neurons (450 total) was trained via the Kohonen algorithm over 1000 iterations. The component planes facilitated a visual assessment of spatial associations and co-occurrence trends among the analyzed variables.

### Two-dimensional kernel density estimation (2D KDE)

A 2D KDE plot was used to visualize the joint distribution of magnetic susceptibility and PAH concentrations. Before analysis, both magnetic susceptibility and ∑PAH concentration values were standardized via Z-score transformation (mean = 0, standard deviation = 1) to account for differences in measurement scales and enable direct comparisons of the variables. The 2D kernel density estimation and visualization were performed via Origin Graph 2019 software.

### PAH ecological risk evaluation

The ecological risk of PAH in road dust was assessed via a method adapted from sediment quality guidelines (SQGs) (Long et al., 1995; McCready et al., 2006; Wang et al., 2020), which were originally developed for evaluating PAH contamination in river sediments. The potential ecological risks posed by PAHs were assessed by comparing their concentrations to the effect range-median (ERM) values provided in Table S3. ERM values used for the ecological risk assessment were mainly taken from Long et al. (1995). For compounds without specific individual ERM values in the original publication (e.g., BghiP and IP), the summary value for high-molecular-weight PAHs (9.6 mg/kg) was applied. For BbF, BkF, and DBA, literature-derived values from Ramzi et al. (2017) and Han et al. (2021) were used. To evaluate the environmental impact of different PAH homologs, the mean ERM quotient (MERM-Q) was calculated, reflecting the average ratio of individual PAH concentrations to their corresponding ERM values. This index helps categorize ecological risk levels from negligible to high. The MERM-Q was computed according to Eq. (2):

2 $$\mathrm{M}\mathrm{E}\mathrm{R}\mathrm{M}-\mathrm{Q}=\left[ \frac{{C}_{i}}{{ERM}_{i}}\right]/ \mathrm{N}$$

where C_i_ represents the measured concentration of the individual PAH component, ERM_i_ represents the ERM value for each individual PAH and N denotes the total number of PAHs considered. A MERM-Q value of 1.5 or higher indicates high ecological risk; values between 0.5 and 1.5 suggest moderate risk; values from 0.1 to below 0.5 reflect low risk; and values under 0.1 are considered to indicate negligible ecological risk (Long et al., 1995; Lin et al., 2017; Wu, 2019a, 2019b; Zhang et al., 2021).

## Results and discussion

### PAH concentration and chemical composition

**Fig. 1:**
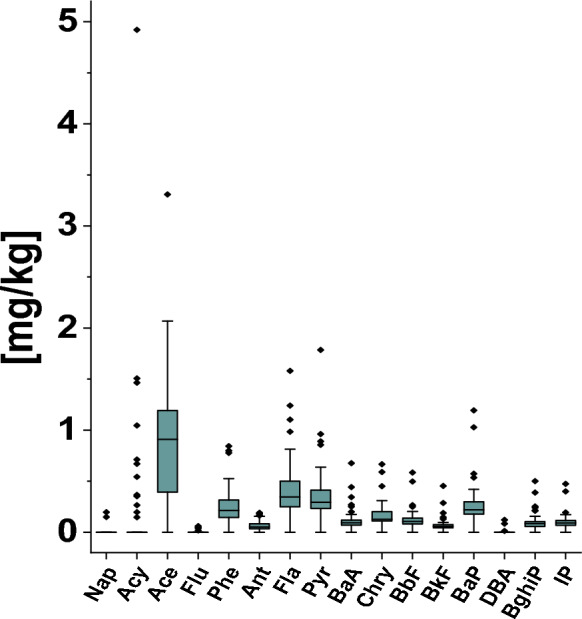
Whisker plots illustrating the concentration ranges of 16 PAH detected in road dust. Whiskers contain the maximum value, mean value, and minimum value. The black horizontal lines represent the means. Triangles represent individual concentration values for samples collected at specific sampling locations

The total concentrations of the 16 priority PAHs (Table S2 in the Supplementary Materials) in the analyzed road dust samples ranged from < LOQ to 12**.**0 mg/kg, with a mean value of 3**.**5 mg/kg and a standard deviation of 2.17 mg/kg. The lowest value (all PAH < LOQ) was recorded in a sample in which no PAH was detected. This sample originated from an unpaved residential access road located in the southeastern part of the city. A whisker plot (Fig. 1) was constructed to represent the concentration distributions of 16 individual PAHs quantified in road dust samples collected from the study area. The analysis of individual PAH concentrations revealed that Acy, Ace, Pyr, Fla, and BaP exhibited the highest concentration ranges, with maximum values exceeding 1.0 mg/kg. Specifically, the highest peak concentration was recorded for Acy (4.92 mg/kg; mean 0.237 ± 0.703 mg/kg), followed by Ace (3.31 mg/kg; mean 0.850 ± 0.663 mg/kg), Pyr (1.79 mg/kg; mean 0.354 ± 0.267 mg/kg), Fla (1.58 mg/kg; mean 0.414 ± 0.282 mg/kg), and BaP (1.19 mg/kg; mean 0.265 ± 0.195 mg/kg). In contrast, Phe reached a maximum of only 0.844 mg/kg (mean 0.256 ± 0.173 mg/kg), and Flu remained at negligible levels (mean 0.00347 mg/kg), confirming that these compounds did not exceed the 1.0 mg/kg threshold. Acenaphthylene (Acy) concentrations ranged from < LOQ to 4.92 mg/kg, with the peak value standing out clearly from the rest of the data. This compound was detected in 12 of the 57 samples, indicating a limited but locally elevated occurrence. The unusually high concentrations of Acy (mean: 0.237 ± 0.703 mg/kg) and acenaphthene (Ace) (max.3.31 mg/kg, 0.850 ± 0.663 mg/kg) constitute a distinctive feature of the road dust in Warsaw. In contrast to many international studies, where higher-molecular-weight (4–6 ring) PAHs from traffic combustion typically dominate (Ha et al., 2012; Hishamuddin et al., 2023; Lee & Dong, 2010), the marked enrichment in these low-molecular-weight (2–3 ring) compounds points to a mixed petrogenic and low-stack pyrogenic signature.

Petrogenic contributions likely originate from vehicle-related oil leaks, spills of lubricating oils and diesel fuel, as well as abrasion and weathering of asphalt pavement (Boonyatumanond et al., 2007; Zabarmawi & Kenig, 2024). In the specific urban context of Poland, an additional important source is residential low-emission heating using coal and biomass, which releases significant amounts of lighter PAHs during incomplete combustion, particularly during the heating season (Siudek et al., 2022).

Classification of sampling sites based on official CEEB data (2023) revealed substantial differences in the share of off-grid heating systems across districts, with the highest proportions observed in right-bank districts (e.g., Wesoła 99.4%, Wawer 99.2%, Rembertów 98.0%, Białołęka 95.8%). Due to atmospheric transport and mixing within the urban canopy, emissions from these areas can influence road dust composition even in more central, grid-connected districts. This distinctive PAH profile in Warsaw may additionally be enhanced by local factors such as an older vehicle fleet with higher rates of oil leaks, intensive asphalt wear, frequent use of de-icing agents, and specific street cleaning practices, all of which can promote the accumulation and release of low-molecular-weight PAHs into road dust.

Fluorene and phenanthrene were also frequently detected at moderate to high concentrations, contributing significantly to the overall PAH profile in multiple samples. In contrast, compounds such as naphthalene were rarely detected. Naphthalene appeared in only two samples, with concentrations of 0.149 and 0.196 mg/kg, suggesting a negligible contribution to the total PAH content. Several other compounds, including benzo[a]pyrene (BaP), benzo[g,h, i]perylene (BghiP), and indeno[1,2,3-cd]pyrene (IP), occurred at consistently low concentrations across nearly all samples, typically not exceeding 0.1 mg/kg. The highest value of the sum of 16 PAHs (12.0 mg/kg) was recorded at a location with relatively high traffic intensity (~ 18,000 vehicles/day) (WBR, 2023) (Table S2 in Supplementary Materials). Limited dispersion of contaminants, resulting from the presence of tall buildings, a relatively confined urban structure (Wang et al., 2011), and reduced sunlight exposure (Gbeddy et al., 2021; Tobiszewski & Namieśnik, 2012), are considered the key factors contributing to the elevated PAH concentrations at this location.

Most studies report PAH levels based either on bulk road dust samples or on specific grain size fractions that differ from ours-most commonly the very fine fraction (< 63 μm). A direct comparison of PAH concentrations with existing literature is inherently challenging, as researchers frequently employ different methodological approaches. Most studies report PAH levels either for bulk road dust samples or for specific grain-size fractions that differ from ours—most commonly the very fine fraction (< 75 or < 63 μm). For instance, in the industrial city of Ulsan, South Korea, Dong and Lee (2009) reported that total PAH concentrations at individual sampling sites ranged from 11.8 mg/kg to as high as 245.1 mg/kg. The authors emphasized that the highest concentrations were found in particles smaller than 180 μm (particularly in the < 75 μm and 75–180 μm fractions), while larger particles (> 180 μm) contained significantly lower PAH levels. Similarly, Ha et al. (2012) investigated the particle-size distribution of PAHs in road dust from Masan, South Korea, and found that ∑16PAH concentrations in heavy traffic areas ranged from 0.45 to 12.0 mg/kg. Notably, they observed a bimodal distribution pattern in these areas, with significant peaks in both the fine fraction (< 63 μm) and the medium-sized fraction (300–1180 μm).

In a broader geographical and methodological context, the ∑16PAH concentrations observed in this study were notably higher than those reported for several other urban environments. Generally, the mean concentration of ∑16PAH in the analyzed road dust samples in this study was one order higher than those reported in Tokyo, Japan (mean ∑12PAH, 0.3 mg/kg; Khanal et al., 2018), Saudi Arabia (mean ∑15PAH, 0.3 mg/kg; Shabbaj et al., 2018 and mean ∑16PAH, 0.43 mg/kg; Alghamdi et al., 2021), Asansol, India (mean ∑16PAH, 0.5 mg/kg; Gope et al., 2018), Jalalabad, Afghanistan (∑17PAH, 0.29 mg/kg; Khpalwak et al., 2019), Iran (with concentrations varying from 0.3 to 2.2 mg/kg; Abbasi & Keshavarzi, 2019; Najmeddin and Keshavarzi, 2019; Davoudi et al., 2021), Vietnam (∑19PAH = 1.7 mg/kg; Mon et al., 2020), and Huanggang, China (1.8 mg/kg; Liu et al., 2019), indicating a relatively high level of PAH contamination in the studied area. The mean PAH level in this study was comparable to that in Chinese cities and provinces such as Hubei Province (mean ∑16PAH: 4.43 mg/kg; Zhang et al., 2016), Qingyang (mean ∑16PAH: 3.0 mg/kg; Wu et al., 2020), Shanghai (mean ∑16PAH: 3.92 mg/kg; Wang et al., 2021), Huainan (mean ∑16PAH: 4.1 mg/kg; Xu et al., 2022) and Chengdu (mean ∑16PAH: 4.8 mg/kg; Li et al., 2021). The exception is Tianjin, China, where the average ∑16PAH concentration in urban street dust was significantly higher at 8 mg/kg (Yu et al., 2014).

**Fig. 2:**
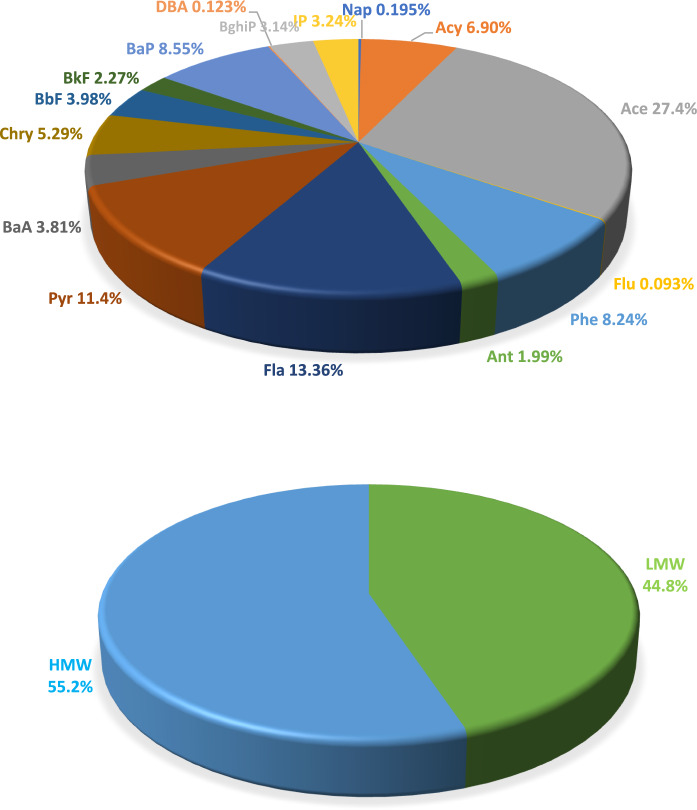
Relative contributions of individual PAHs to the total PAH concentration in road dust. Bottom chart: Contribution of individual PAHs to total PAH concentrations

On the basis of the number of aromatic rings, the 16 PAHs were categorized into two groups: LMW-PAHs with 2–3 rings and HMW-PAHs with 4–7 rings (Fig. 2, Table S4). LMW PAHs tend to be more volatile and mobile in the environment, whereas HMW PAHs are more persistent, less soluble, and more likely to adsorb onto particles and accumulate in sediments or dust.

Figure 1 presents the relative contributions of 16 individual PAHs to the total PAH concentration in road dust samples collected from urban streets. These results indicate that the PAH profile is dominated by low- and medium-molecular-weight compounds. Ace was the most abundant, contributing 27.4% of the total concentration, followed by Fla (13.4%) and Pyr (11.4%). Ace made the greatest contribution among the PAHs in road dust from Tehran (Iran) (Mihankhah et al., 2020) and Qom city (Iran) (Davoudi et al., 2021).

The individual high-molecular-weight PAHs (HMW-PAHs) showed varying contributions to the total mass. Among them, Fla (13.4%) and Pyr (11.4%) were the most abundant, being the only HMW compounds to exceed the 10% threshold. The remaining HMW PAHs each accounted for less than 10% of the total mass, specifically: BaP (8.56%), Chr (5.29%), BbF (3.98%), BaA (3.81%), IP (3.24%), BghiP (3.13%), and BkF (2.27%), while DBA showed the smallest individual share at 0.123%.

Collectively, HMW-PAHs accounted for 55.2% of the total PAH mass, reflecting the predominant influence of mobile emission sources-particularly vehicular traffic-over stationary combustion activities. These compounds form during high-temperature combustion and tend to adsorb onto fine particles, which may be underrepresented in the sampled road dust fraction or subject to distinct atmospheric deposition dynamics. LMW-PAHs constituted the remaining 44.8%, and although they accumulate in the environment, their persistence in road dust is lower than that of HMW-PAHs due to higher volatility, chemical reactivity, and susceptibility to photodegradation (Teixeira et al., 2012; Friedman et al., 2014). Their elevated proportions may additionally reflect more recent deposition linked to their greater mobility.

### Source identification

#### Diagnostic ratios

Figure S3 (Supplementary Materials) presents cross-plots of selected isomeric ratios: IP/(IP + BghiP) versus BaA/(BaA + Chr), IP/(IP + BghiP) versus Fla/(Fla + Pyr), BaA/(BaA + Chr) versus Fla/(Fla + Pyr), and Ant/(Ant + Phe) versus Fla/(Fla + Pyr). This multi-plot approach has been widely used in previous studies to distinguish pyrogenic from petrogenic PAH sources (Stogiannidis & Laane, 2015; Tobiszewski & Namieśnik, 2012; Yunker et al., 2002).

The four isomeric cross-plots (Figure S3) collectively indicate a dominant pyrogenic origin of PAHs in the studied metropolitan road dust. In the BaA/(BaA + Chr) versus IP/(IP + BghiP) plot, most samples fall within the vehicular emission and mixed sources fields, with IP ratios mainly between 0.4 and 0.6 and BaA/(BaA + Chr) values above 0.35, pointing to high-temperature combustion of gasoline and diesel fuels (Yunker et al., 2002; Zhang et al., 2019). The Fla/(Fla + Pyr) versus IP/(IP + BghiP) diagram further supports traffic as the main contributor, while a subgroup of points in the wood/coal/grass combustion zone suggests an additional influence from solid fuel burning (Hishamuddin et al., 2023; Li et al., 2025). The Fla/(Fla + Pyr) versus BaA/(BaA + Chr) plot shows most points in the vehicular and petroleum/coal combustion areas, with very few samples in the strictly petrogenic zone, indicating only minor contribution from unburned petroleum or oil spills (Zhang et al., 2008, 2019). Finally, the Ant/(Ant + Phe) versus Fla/(Fla + Pyr) cross-plot confirms the predominantly pyrogenic character, with the majority of samples clustering in the high-temperature combustion domain (Ant/(Ant + Phe) > 0.1 and Fla/(Fla + Pyr) > 0.4). This is consistent with urban road dust profiles where vehicular exhaust coexists with coal and biomass combustion (Hishamuddin et al., 2023; Jahedi et al., 2026; Zhang et al., 2008). Overall, the integrated diagnostic ratio analysis demonstrates that high-temperature pyrogenic processes, primarily traffic-related, dominate the PAH burden, with only limited petrogenic inputs (Hishamuddin et al., 2023; Jahedi et al., 2026; Yunker et al., 2002; Zhang et al., 2019). This pattern aligns well with findings from other global urban studies (Ghanavati et al., 2019; Yusuf et al., 2022).

In the present study, Phe/Ant ratios were mostly below 10 (mean ± SD = 3.47 ± 2.64, range 0–15.5), with only one sample exceeding this value, further supporting a primarily pyrogenic origin linked to fossil fuel combustion and biomass burning (Katsoyiannis et al., 2011; Tobiszewski & Namieśnik, 2012). The BaP/BghiP ratios ranged from 0 to 6.87 (mean ± SD = 2.66 ± 1.46); only three samples were below 0.6, confirming that traffic-related emissions constitute the dominant PAH source in the area (Yunker et al., 2002; Zhang et al., 2019). Similarly, Pyr/BaP ratios averaged 1.231 ± 0.459, with most values close to 1, indicating a prevailing influence of gasoline combustion (Yunker et al., 2002). BaA/Chr ratios exceeded 0.5 in all but two samples, reinforcing the importance of combustion processes (Tobiszewski & Namieśnik, 2012; Yunker et al., 2002).

PAHs from combustion processes reflect the temperature and efficiency of burning: low-to-moderate temperatures generate more low-molecular-weight and alkylated compounds, while high-temperature combustion produces heavier PAH species. In contrast, petrogenic sources (crude oil or refined petroleum) are enriched in alkylated and lighter (2–3 ring) PAHs (Mai et al., 2003). The LMW/HMW ratio is commonly applied to differentiate these origins. In the Warsaw road dust samples, LMW/HMW ratios ranged from 0.177 to 3.38 (mean = 0.923). Most samples fell below 1.3, indicating predominantly pyrogenic to intermediate sources, with only three samples (≈5.26%) exceeding 2.0, revealing a distinct petrogenic signal (Table S4).

#### Principal component analysis (PCA) and hierarchical cluster analysis (HCA)

**Fig. 3:**
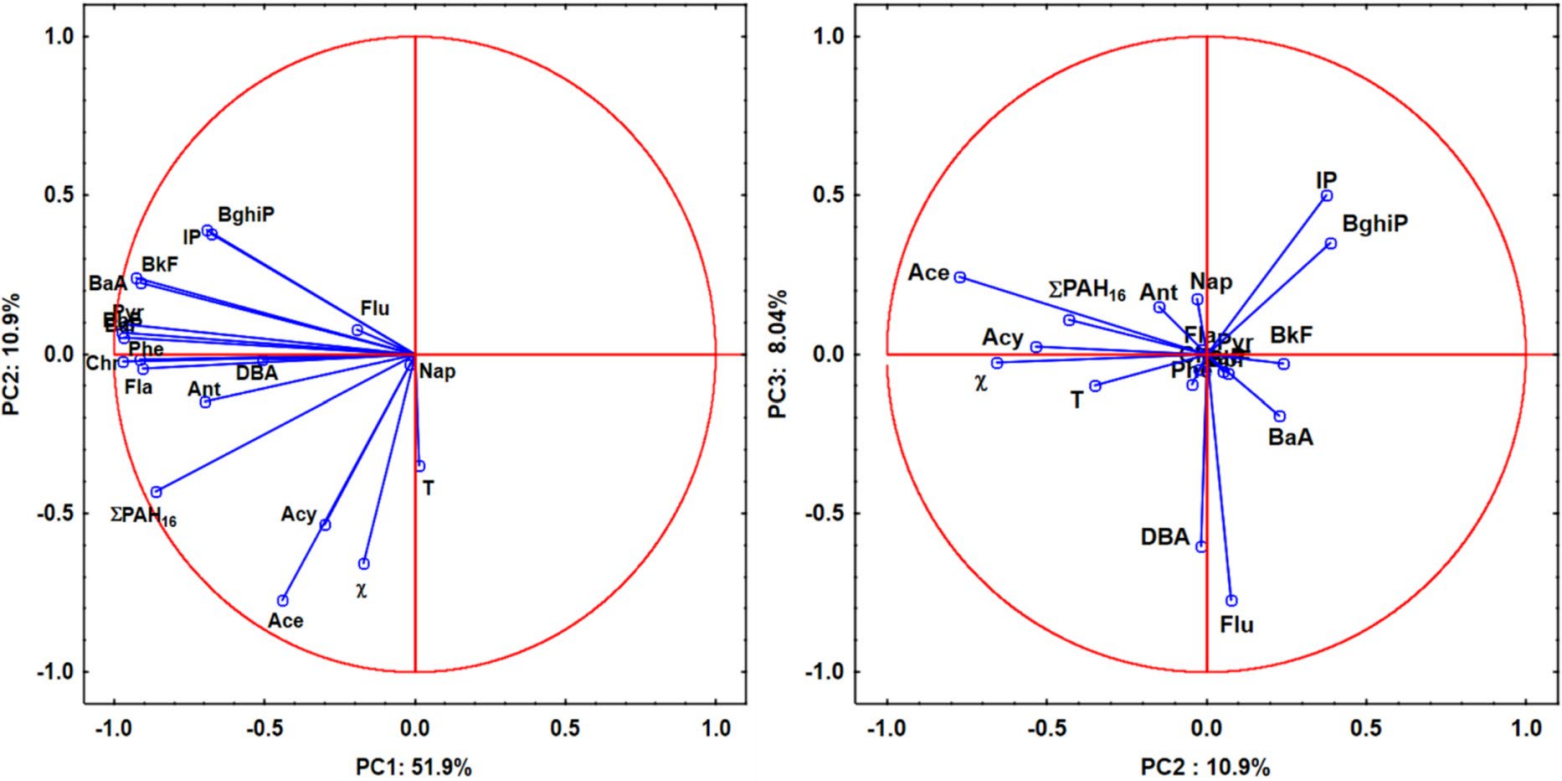
Principal Component Analysis (PCA) biplots showing the relationships between magnetic susceptibility (χ), total PAH concentrations (∑PAH16), traffic intensity (T), and individual PAH compounds in road dust. Left panel: projection on the PC1–PC2 factor plane. Right panel: projection on the PC2–PC3 factor plane. Abbreviations: LMW PAHs (Low Molecular Weight): Nap–Naphthalene, Acy–Acenaphthylene, Ace–Acenaphthene, Flu–Fluorene, Phe–Phenanthrene, Ant–Anthracene; HMW PAHs (High Molecular Weight): Fla–Fluoranthene, Pyr–Pyrene, BaA–Benz[a]anthracene, Chr–Chrysene, BbF–Benzo[b]fluoranthene, BkF–Benzo[k]fluoranthene, BaP–Benzo[a]pyrene, DBA–Dibenz[a,h]anthracene, BghiP–Benzo[ghi]perylene, IP–Indeno[1,2,3-cd]pyrene; χ–mass-specific magnetic susceptibility; T–traffic intensity

The PCA biplots (Fig. 3) illustrate the relationships between magnetic susceptibility (χ), traffic intensity (T), individual PAH concentrations, and the sum of PAHs (∑PAH16), projected onto the first three principal components. The first two components (PC1 and PC2) explain 62.8% of the total variance (51.9 and 10.9%, respectively), while the combination of PC2 and PC3 accounts for 18.9% (10.904 and 8.04%).

In the left PCA biplot (PC1 vs. PC2), most 4- to 6-ring PAHs, such as BaP, BbF, BkF, Chr, BghiP, IP, Pyr, BaA, and Fla, show strong negative loadings on PC1, indicating their dominant contribution to this axis. These high molecular weight (HMW) PAHs are typically associated with pyrogenic sources. In contrast, Ace, Acy, and χ exhibit negative loadings on PC2, suggesting their contribution to this dimension and a possible link with petrogenic or lighter PAH sources. Major pyrogenic sources of Ant and Phe include the combustion of municipal solid waste, incomplete fossil fuel combustion in vehicles, as well as wood and biomass burning (Jiao et al., 2017). IP is a key marker of petroleum combustion and vehicle exhaust emissions (Sadiktsis et al., 2012). BaA and Chr are primarily emitted from diesel combustion (Jiang et al., 2016), whereas BbF is mainly associated with fossil fuel combustion (Jia et al., 2017). Consequently, PC1 primarily reflects combustion-related emissions from municipal waste, fossil fuels, and biomass.

In the right PCA plot (PC2 vs. PC3), Ace, Acy, χ, T, and ∑PAH16 display strong negative loadings on PC2. The close grouping of traffic intensity (T) with magnetic susceptibility (χ) and total PAH content along this axis confirms that vehicle-related activities are the primary drivers of both magnetic and organic contamination in the studied road dust. In contrast, Flu and DBA load significantly negatively on PC3, being clearly separated from the LMW group. High molecular weight (HMW) compounds, such as IP and BghiP, are positioned in the upper right quadrant with positive loadings on both PC2 and PC3. LMW PAHs, such as Acy, Ace, Flu, and Ant, are typically associated with fossil fuels (Oliva et al., 2015), while Flu is specifically linked to low-temperature, incomplete combustion of fuel residues (Yunker et al., 2022).

A similar pattern, with HMW PAHs (pyrogenic and mixed origin) loading on PC1 and LMW PAHs (often associated with petrogenic or specific combustion sources) loading on PC2, has also been observed in studies from Dalian, China (Wang et al., 2007), Shenzhen, China (Shi et al., 2024), Sydney, Australia (Nguyen et al., 2014), Tianjin, China (Wu et al., 2022; Yu et al., 2014), and Ulsan, Korea (Lee & Dong, 2010).

Figure S4 (Supplementary Materials) presents the dendrogram generated via Ward’s method and the Euclidean distance. The first cluster consists of Nap, Flu, DBA, Ant, BkF, BghiP, and IP. These compounds cluster together at a small distance of approximately 1, indicating strong similarity. This cluster mainly includes HMW PAHs, which are typically produced during the incomplete combustion of fossil fuels and industrial emissions. Many of these compounds, such as DBA, BKF, BghiP, and IP, are known for their carcinogenic and mutagenic properties. The second cluster included BaA, BbF, Chr, Phe, BaP, Fla, and Pyr. These PAHs join at a slightly larger distance of approximately 2, suggesting moderate similarity. This group contains compounds with varying molecular weights but is generally associated with combustion products, coal tar, and fossil fuels. Many in this cluster, such as BaP, are potent carcinogens and important environmental indicators. The third cluster is formed by Ace and Acy, which cluster together at a larger distance of approximately 5–6. These low-molecular-weight PAHs are less toxic but are useful as tracers of specific contamination sources, such as petroleum products and lower-temperature combustion. Overall, the clustering reflects the chemical properties and typical sources of these PAHs: lower-molecular-weight, more volatile compounds separate from heavier, more toxic PAHs, which are produced mainly by high-temperature combustion and industrial activities.

#### Self-organizing map (SOM)-Kohonen map

Diagnostic ratios provided a classical approach to distinguish between pyrogenic and petrogenic sources, while Principal Component Analysis (PCA) identified the main linear relationships and variance structures among PAH compounds, traffic intensity, and magnetic susceptibility. Hierarchical Cluster Analysis (HCA) grouped individual PAHs according to their chemical similarity and shared origins. However, these methods are primarily linear or based on pairwise comparisons.

To complement these approaches and explore non-linear relationships in the high-dimensional dataset, a Self-Organizing Map (SOM) was applied. SOM is an unsupervised artificial neural network technique that projects multivariate data onto a two-dimensional grid while preserving the topological relationships between samples and variables.

**Fig. 4:**
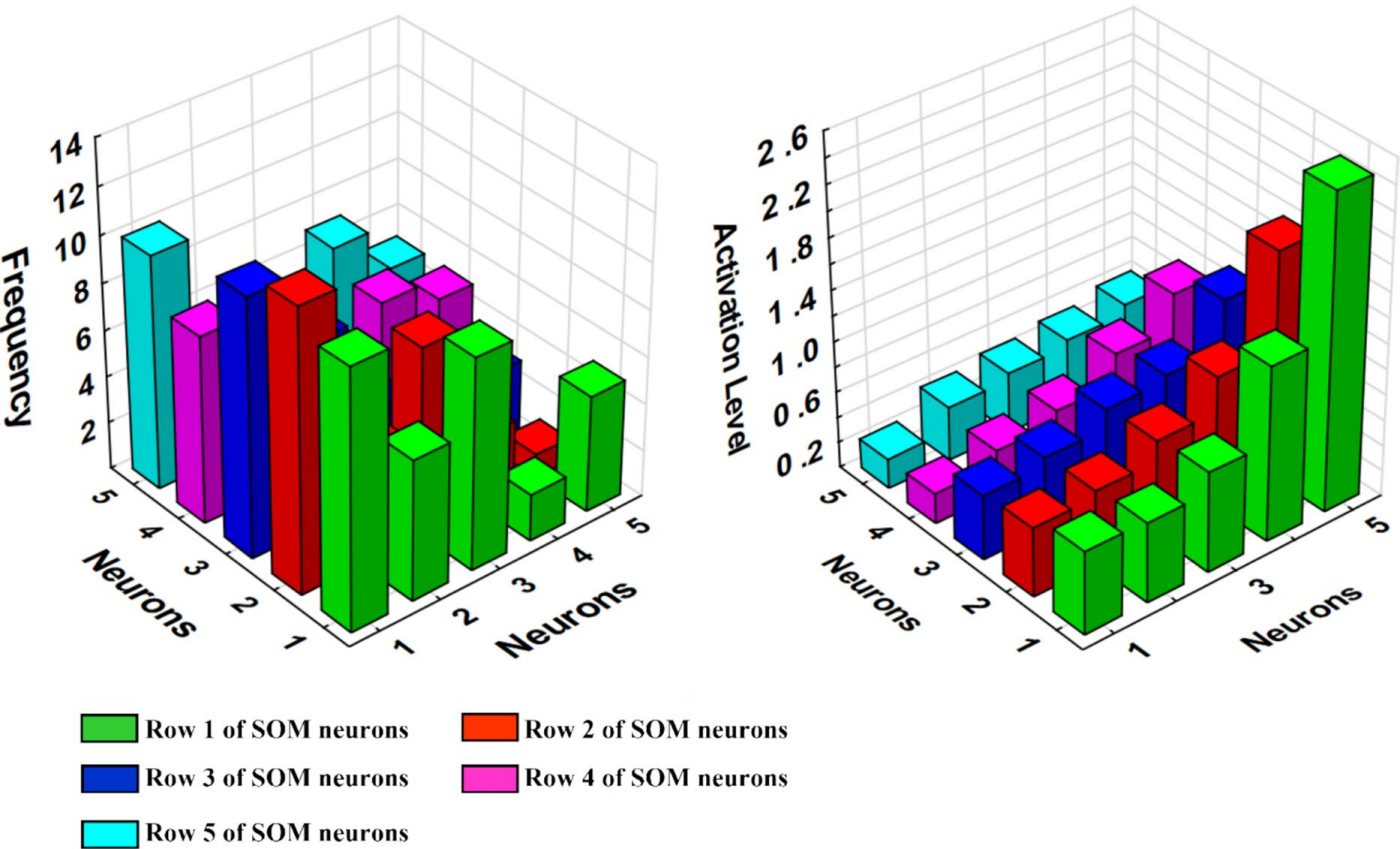
Visualization of the 5 × 5 Self-Organizing Map (SOM) results. Left panel: Distribution of sample frequencies assigned to each neuron; the vertical axis represents the number of road dust samples mapped to specific neuron coordinates. Right panel: Activation levels of SOM neurons visualized as a 3D bar chart; the vertical axis indicates the degree of similarity (activation level) between the input vectors and the neuron weight vectors. Note: Color coding is used to distinguish different neuron clusters, as detailed in the legend and the main text

The trained 5 × 5 SOM demonstrated good performance, with training, testing, and validation errors of 0.0917, 0.1326, and 0.0979, respectively. The frequency of samples assigned to each neuron (Fig. 4) ranged from 1 to 12, with the highest counts recorded in neurons (1,1) and (2,1). Neuron activation levels ranged from 0.24 to 2.47, reaching a maximum of 2.47 at neuron (1,5).

Analysis of neuron weight vectors (Table S5 in Supplementary Materials) revealed three distinct patterns. Neuron (1,1) was characterized by very low weights across most variables, representing background sites with minimal contamination. Neuron (2,5) showed moderate magnetic susceptibility and traffic intensity, but elevated weights for low-molecular-weight PAHs (Nap, Acy, Ace, Flu), suggesting petrogenic inputs or low-temperature combustion sources. In contrast, neuron (4,3) exhibited the highest magnetic susceptibility together with high traffic intensity and elevated HMW-PAH concentrations (BaP, BbF, BkF, IP), indicating intense traffic-related pyrogenic contamination.

Compared to diagnostic ratios, PCA, and HCA, the SOM provided additional nonlinear insights through the analysis of neuron weight vectors. While the linear methods effectively demonstrated the overall dominance of traffic-related pyrogenic sources, the SOM enabled a clearer distinction between different contamination regimes. Specifically, it separated a high-traffic domain strongly associated with HMW-PAHs and magnetic susceptibility from areas with increased LMW-PAH contributions at moderate traffic intensity and χ. This separation highlighted the coexistence of the dominant vehicular pyrogenic source with subordinate petrogenic and low-temperature combustion inputs—a nuance that was less distinctly resolved by the previously applied methods.

### Weight-normalized magnetic susceptibility (χ_WN_) and weight normalized PAH (∑PAHWN)

Figure S5 shows the relationships between magnetic susceptibility (χ) and the concentrations of total PAHs (∑ PAHs, left panel) and ∑ HMW PAHs (right panel) via Z-score standardized values. In both plots, a positive correlation is visible, particularly concentrated in the lower left quadrant, indicating that samples with low χ values also tend to have lower PAH concentrations, and vice versa.

The density gradients in both panels highlight that most data points are clustered around slightly negative to near-zero Z-scores, suggesting that the majority of the samples have χ and PAH concentrations near the mean. However, there are also several outlier points in the upper-right quadrant, which are particularly prominent in the ∑ HMW PAH plot, indicating that in some samples, elevated levels of magnetic susceptibility are associated with higher concentrations of HMW PAH.

**Fig. 5:**
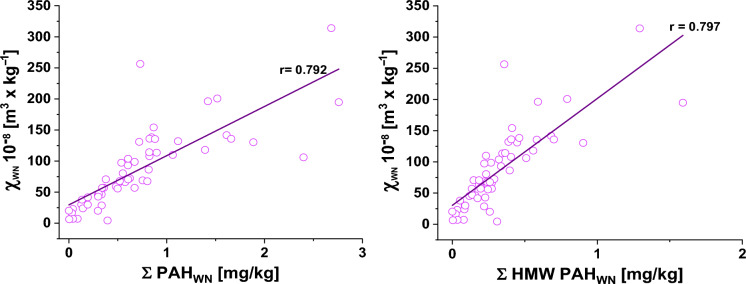
Correlation between weight-normalized magnetic susceptibility (χ_WN_) and PAH concentration. Right panel*:* correlation with HMW-PAHs (∑HMW-PAH_WN_). Left panel*:* correlation with total PAH (∑PAH_WN_)

Figure 5 presents scatter plots with linear regression lines illustrating the relationship between the weighted normalized magnetic susceptibility (χ_WN_) and the concentration of PAH. Owing to the variability in the proportion of the fine fraction, ranging from ~ 2.84% to ~ 50.4% (Figure S2 in Supplementary Materials) of the total sample, weight-normalized magnetic susceptibility (χ_WN_), ∑PAH_WN**,**_ and ∑ HMW PAH_WN_ were introduced. This parameter was calculated by multiplying the mass-specific magnetic susceptibility (χ), ∑PAH, and ∑ HMW PAH by the percentage content of the fine fraction in each bulk sample.

The left and right panels display the correlation between magnetic susceptibility χ_WN_ and ∑PAH_WN_. The Pearson correlation coefficient is approximately r = 0.792, with a coefficient of determination R^2^ of approximately 0.628, explaining 62.8% of the variation.

For ∑PAH_WN,_ the linear regression equation is as follows:

$$\sum PAH_{WN} = \left( {29.7 \pm 7.83} \right) + \left( {79.2 \pm 8.22} \right) \times \chi_{WN}$$

The right panel shows the correlations with ∑ HMW PAH_WN_ and χ_WN._ The linear regression analysis between ∑ HMW PAH_WN_ and χ_WN_ resulted in the following equation:

$$\sum HMW \, PAH_{WN} = \left( {30.4 \pm 7.66} \right) + \left( {171 \pm 17.5} \right) \times \chi_{WN}$$

The Pearson correlation coefficient of r = 0.797 indicates a strong positive correlation between ∑ HMW PAH_WN_ and χ_WN_. The coefficient of determination, R^2^ = 0.635, shows that approximately 63.5% of the variance in ∑ HMW PAH_WN_ can be explained by χ_WN_.

The consistency of these values supports the use of χ_WN_ as a reliable magnetic proxy for assessing PAH contamination. The strength of the observed correlations suggests a shared anthropogenic source for both magnetic carriers and PAHs, most likely emissions from combustion-related activities such as vehicle exhaust and industrial processes.

To the best of our knowledge, no previous studies have developed regression models that directly relate magnetic susceptibility to total or high-molecular-weight PAH concentrations in urban dust samples. Therefore, the models presented here represent a novel contribution to the field, providing practical tools for estimating PAH contamination on the basis of magnetic measurements.

Although χ_WN_ explained a substantial portion of the variance in PAH concentrations (R^2^ = 0.628 for ∑PAH_WN_ and R^2^ = 0.635 for ∑HMW PAH_WN_), 36.5–37.2% of the variance remained unexplained. This residual variability reflects the contrasting post-depositional behaviour of anthropogenic magnetic particles and PAHs in the dynamic urban road dust matrix. Anthropogenic magnetic particles, primarily technogenic magnetite, are relatively stable under urban surface conditions and contribute directly to the measured magnetic susceptibility with limited post-depositional alteration (Yang et al., 2020; Zhang et al., 2021). In contrast, PAH concentrations are strongly modified by multiple transformation and removal processes. These include photodegradation (Gbeddy et al., 2021), volatilization of low-molecular-weight compounds (Majumdar et al., 2012), microbial biodegradation (Johnsen & Karlson, 2005), chemical oxidation, reduction, and hydrolysis influenced by surface-bound metals such as Fe(III) (Gbeddy et al., 2020), and physical removal through stormwater wash-off, aeolian resuspension, and traffic-induced turbulence (Liu et al., 2017). As a result, the final amount of PAHs accumulated in road dust is considerably more variable and dynamic than the more stable magnetic signal.

### MERM-Q ecological risk assessment

**Fig. 6:**
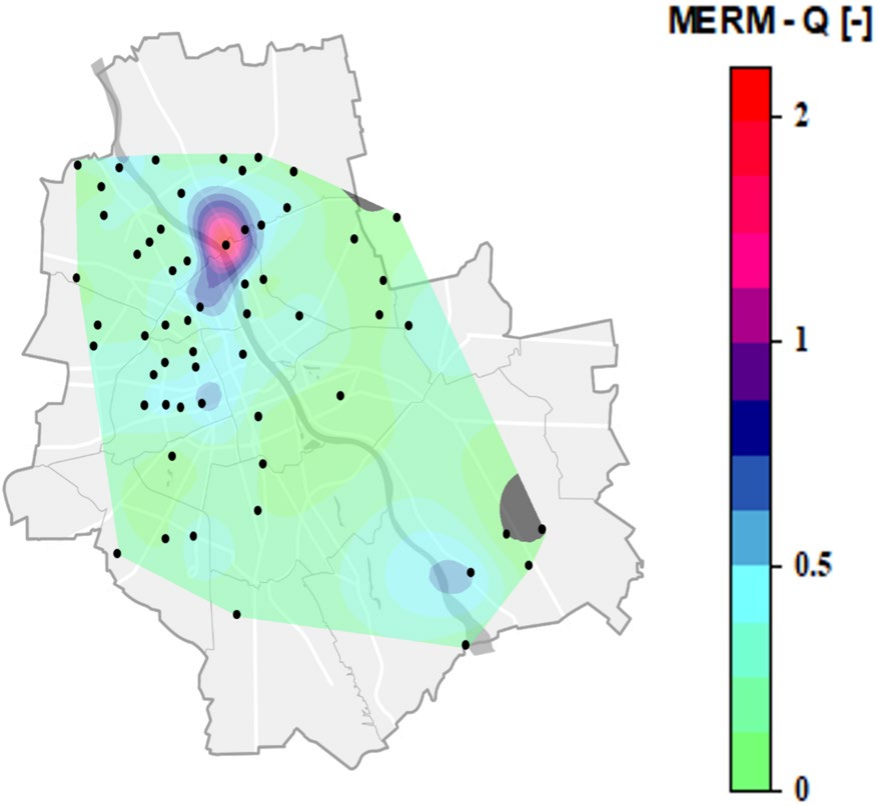
Spatial distribution of the MERM-Q values of the ecological risk indicators calculated for PAHs in road dust collected across Warsaw

As there may be different ecological effects of simultaneous exposure to a mixture of PAHs and the ecological risk of an individual PAH may underestimate or overestimate the actual risk of a PAH (Lin et al., 2017), the MERM-Q method was applied to assess the combined ecological risks of the 16 parent PAHs in road dust. The map (Fig. 6) presents the spatial variation in the MERM-Q index based on the concentration and toxic equivalency of PAHs. The spatial distribution suggests that elevated MERM-Q values are associated with regions of higher traffic intensity. Among the 57 road dust samples analyzed, 5 presented medium ecological risk (MERM-Q between 0.5 and 1.5), 7 presented negligible risk (MERM-Q < 0.1), and only one sample posed high risk with a value of MERM-Q > 1.5, while the majority of the 44 samples fell into the low-risk category (MERM-Q between 0.1 and 0.5). These findings are in agreement with those of Nematollahi et al. (2021), who reported that road dust from a location in Yazd, a central capital city in Iran, posed potential adverse effects (0.1 < MERM-Q ≤ 0.5), likely due to its proximity to oil depots, whereas the remaining samples indicated low ecological risk (MERM-Q < 0.1). The highest MERM-Q values were recorded in the northeastern part of the study area at the same location that exhibited the highest magnetic susceptibility (1066 × 10^−8^ m^3^/kg), which is associated with heavy traffic and high vehicle intensity. In South Korea, Moon et al. (2024) reported low to medium ecological risk levels for urban areas such as Gunsan (with a MERM-Q value of 0.218), whereas other sites in the country, including Ulsan and Busan, presented lower values below 0.1, indicating minimal risk. Similar levels of ecological risk were also observed in Chinese cities such as Xiamen and Changchun. In contrast, much higher ecological risk values have been identified in Port Harcourt, Nigeria, where the quotient exceeds 4.0, indicating substantial PAH contamination and potential environmental concern.

### Spatial distribution of PAH

**Fig. 7:**
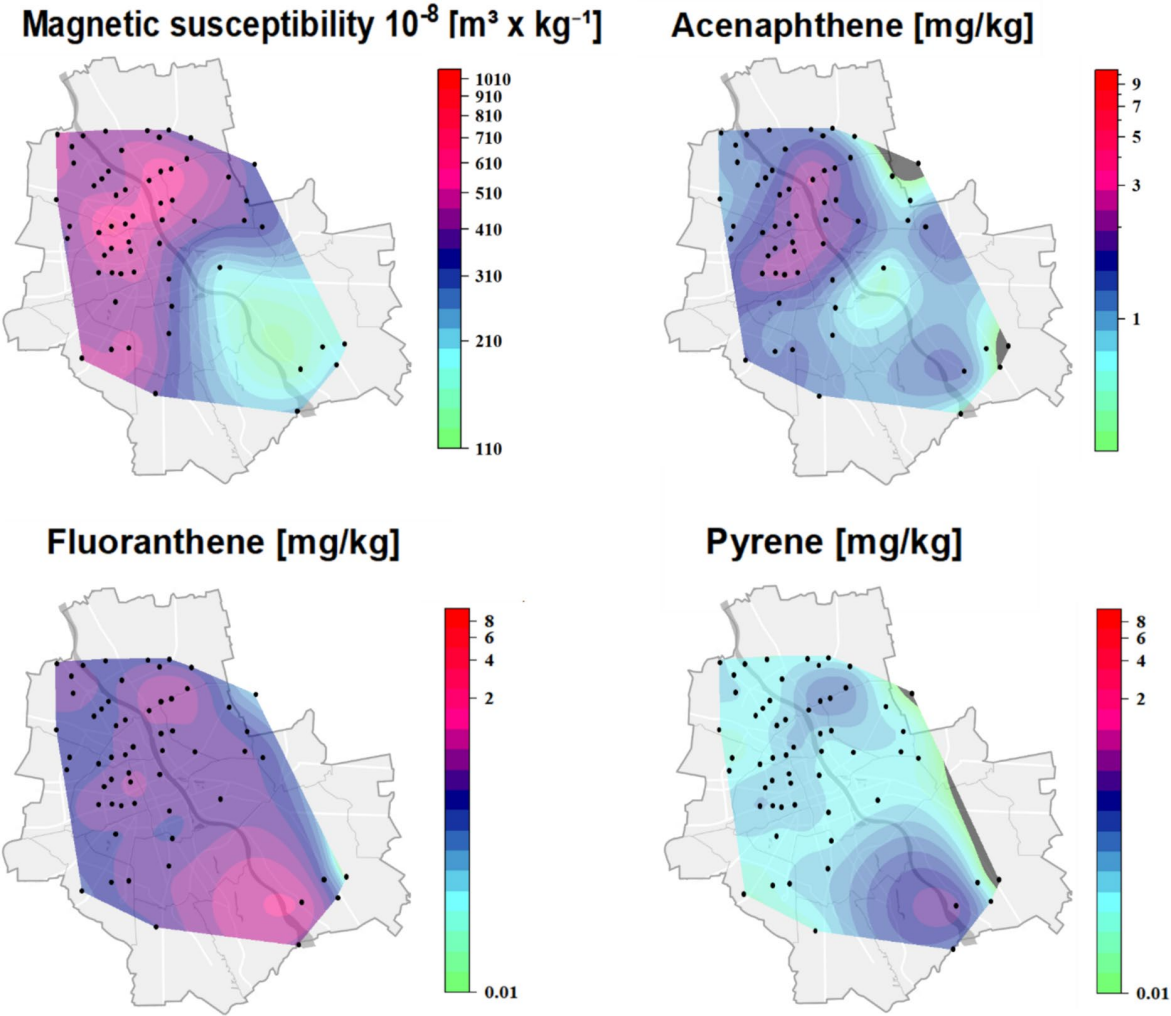
Spatial distribution maps of selected parameters measured in road dust samples collected from Warsaw, Poland. Top left: magnetic susceptibility (10^−8^ m^3^/kg); top right: acenaphthene (mg/kg); bottom left: anthracene (mg/kg); bottom right: pyrene (mg/kg)

The spatial distribution maps (Fig. 7) of magnetic susceptibility and three selected PAH compounds—acenaphthene, anthracene, and pyrene—illustrate notable heterogeneity across the study area. These three PAHs were chosen on the basis of their highest mean concentrations among all quantified compounds in the road dust samples. The highest magnetic susceptibility was observed in the northwestern part of the area, which was dominated by high traffic and dense building development. The magnetic susceptibility (χ) values ranged from 113 × 10^−8^ to 1068 × 10^−8^ m^3^/kg, with a mean value of 429 × 10^−8^ m^3^/kg and a standard deviation of 146 × 10^−8^ m^3^/kg (Table S2 in Supplementary Materials). A similar pattern was observed for acenaphthene, which also presented elevated concentrations in the north, indicating a potential codistribution of magnetic and low-molecular-weight organic contaminants emitted during combustion processes. In contrast, the spatial distribution of anthracene, a heavier PAH, peaked in the southwestern region, suggesting a distinct emission source or deposition mechanism. Pyrene, although selected for its relatively high overall concentration, showed more dispersed and lower intensity patterns, with no clear spatial correlation with magnetic susceptibility. The highest values of χ (1068 × 10^−8^ m^3^/kg) were recorded in the Wola district, at the intersection of Okopowa and Żytnia Streets. This is a major street with tram tracks and heavy traffic, with approximately 35,000 vehicles passing daily (WBR, 2023). These results align with previous studies on the magnetic susceptibility of street dust from Warsaw. Dytłow et al. (2019) reported values in Warsaw ranging from 49 to 1025 × 10^−8^ m^3^/kg. In Shanghai (China), Wang et al. (2019) reported χ values approximately four times greater, between 120 and 4040 × 10^−8^ m^3^/kg, with a mean of 810 × 10^−8^ m^3^/kg, which is consistent with Zhang et al. (2024). Lower values were observed in Fujian coastal cities (21–911 × 10^−8^ m^3^/kg, average 376 × 10^−8^ m^3^/kg; Yang et al., 2020) and on Xiamen Island (25–730 × 10^−8^ m^3^/kg, average 250 × 10^−8^ m^3^/kg; Yang et al., 2021).

## Conclusions

This study demonstrates that weight-normalized magnetic susceptibility (χ_WN_) of the fine fraction (< 0.2 mm) of urban road dust from Warsaw exhibits a strong positive correlation with both total polycyclic aromatic hydrocarbon concentrations (∑16PAH_WN_, r = 0.792, R^2^ = 0.628) and high-molecular-weight PAHs (∑HMW PAH_WN_, r = 0.797, R^2^ ≈ 0.635). These results confirm that χ_WN_ can serve as a reliable, rapid, non-destructive, and cost-effective proxy for screening PAH contamination in road dust.

Source apportionment using multiple diagnostic isomer ratios combined with advanced multivariate techniques (PCA, HCA, and SOM) revealed that PAHs in the studied road dust are predominantly of pyrogenic origin. Vehicular emissions (gasoline and diesel combustion) were identified as the dominant source, with a subordinate contribution from solid fuel combustion (wood, coal, and biomass). Petrogenic inputs from unburned petroleum products were found to be minor.

The highest PAH concentrations and ecological risk (assessed by the mean ERM quotient, MERM-Q) occurred in areas characterized by intense traffic and limited pollutant dispersion, such as dense urban canyons in the city center. Regression models developed in this work provide practical equations for estimating PAH levels directly from magnetic susceptibility measurements.

This research represents the first systematic attempt to apply weight-normalized magnetic susceptibility as a proxy for PAH contamination, specifically in urban road dust. The strong χ_WN_–PAH relationship, together with the complementary use of molecular diagnostic ratios and multivariate statistical tools, offers a comprehensive and efficient framework for urban contamination assessment. The proposed proxy approach has significant potential to optimize sampling strategies, reduce analytical costs, and support large-scale environmental monitoring programs, particularly in cities with limited resources.

The integration of diagnostic ratios, PCA, HCA, and SOM provided complementary insights into PAH sources. While the linear methods confirmed the dominance of traffic-related pyrogenic emissions, the SOM revealed non-linear patterns that highlighted the coexistence of the main vehicular source with subordinate petrogenic and residential combustion inputs.

## Limitations and future research

This study focuses on the relationship between magnetic susceptibility and PAH concentrations in a specific granulometric fraction of urban road dust. Finer particles, which pose greater ecological and health risks because of their respirability, were not analyzed separately. Future studies should include other fractions to better evaluate the proxy potential of magnetic susceptibility. These findings are limited to road dust, whereas the χ–PAH relationship may differ in other media, such as airborne particles, due to the higher volatility and reactivity of PAHs. Geographic variability-differences in contamination sources, urban structure, and climate-may also affect both χ values and PAH levels. Therefore, applying these models elsewhere requires local validation. Although the regression models explain much of the variance, a notable portion remains unexplained, suggesting the influence of additional environmental or chemical factors. Broader studies covering various media and variables are needed to improve the robustness and general applicability of this approach. While this study focused on assessing the ecological risk associated with PAH contamination in road dust, it is important to recognize that these pollutants also pose a direct threat to human health. Given that urban populations are constantly exposed to road dust through inhalation and accidental ingestion, future research should extend this framework to include a comprehensive human health risk assessment (HHRA).

## Environmental implications

This study demonstrated that magnetic susceptibility (χ), particularly its weight-normalized form (χ_WN_), can serve as a fast and cost-effective proxy for assessing PAH contamination in urban road dust. The strong correlation between χ_WN_ and PAH concentrations, particularly those of high-molecular-weight compounds, supports its use in environmental monitoring. In resource-limited settings, this approach can reduce the need for extensive chemical analysis while preserving spatial coverage. This non-destructive method allows repeated use, supports source apportionment, and helps identify contamination hotspots, enabling better-informed urban planning and early responses to ecological risk.

## Supplementary Information

Below is the link to the electronic supplementary material.

Supplementary file1 (DOCX 1912 KB)

## Data Availability

All the data generated or analyzed during this study are included in this published article (and its Supplementary Information files).
