# Supplementary material for: Linking magnetic susceptibility with polycyclic aromatic hydrocarbon concentrations in urban road dust: a proxy-based approach for contamination monitoring
Source: Environ Geochem Health. 2026 May 11;48(8):340. doi: 10.1007/s10653-026-03240-6 (PMC13161304; doi:10.1007/s10653-026-03240-6)
Supplement: Supplementary file 1 — Supplementary file1 (DOCX 1912 KB) [file 10653_2026_3240_MOESM1_ESM.docx]

**Supplementary Materials**

**Linking magnetic susceptibility with polycyclic aromatic hydrocarbon concentrations in urban road dust: A proxy-based approach for contamination monitoring**

**Sylwia Dytłow *^1^Jakub Karasiński ^2^**

^1^ Institute of Geophysics, Polish Academy of Sciences, Ks. Janusza 64, 01-452 Warsaw, Poland

^2^ University of Warsaw, Faculty of Chemistry, Biological and Chemical Research Centre, Warsaw, Poland

0000-0001-6968-6491 Sylwia Dytłow ORCID

*E-mail address of the corresponding Author: Sylwia Dytłow skdytlow@igf.edu.pl

**S1. PAH quantification through chromatographic techniques**

The cumulative PAH concentration was determined by summing the 16 individual compounds included in the analytical standard (Supelco, CRM47940 PAH Calibration Mix), with specific monitored substances detailed in Table S1. To evaluate the levels of specific PAHs, an analytical approach involving high-performance liquid chromatography (HPLC, Agilent 1290) integrated with a series-connected spectrophotometric detector (Agilent G4212B 1260 Diode Array Detector) and a mass spectrometer (Agilent 6460) featuring a triple quadrupole mass analyzer (QQQ) was used. The QQQ system utilized an APCI source (atmospheric pressure chemical ionization) operating in positive ion mode. The initial operations were conducted in scan mode, after which the recording mode was changed to single-ion monitoring to increase the detection sensitivity once the characteristic m/z values for each PAH were established.

For the separation of the 16 PAHs from the standard mixture, a specialized chromatographic column (Phenomenex, Kinetex 3.5 μm PAH, 100 × 2.1 mm) was used. The separation process relied on gradient elution, where 0.1% formic acid (FA, Supelco LC‒MS LiChropur) in water (Supelco LiChrosolv LC‒MS Grade) served as the mobile phase with lower elution strength, whereas 0.1% FA in ACN (Supelco LiChrosolv hypergrade for LC‒MS) acted as the higher-strength phase. The comprehensive parameters for separation and detection are provided in Table S1. During the optimization of the method, five distinct signal acquisition settings were evaluated: 356 nm, 541 nm, and 370 nm (all without reference), as well as 254 nm and 292 nm (both with a 400 nm reference). The configuration yielding the highest sensitivity was identified as 254 nm with a 400 nm reference (Mansouri et al., 2020). Data from both the MS and DAD detectors were subsequently compared, and the elution sequence of specific PAHs was verified via the acquired mass spectra. Given the high degree of correlation between the two detectors, UV detection was exclusively utilized following the completion of method optimization, and all reported findings were derived from UV signals. The identification of particular PAHs was performed by comparing retention times between the samples and the standard. Finally, the extraction performance and integrity of the measurement protocol (including QC and recovery tests) were validated through the analysis of matrix-certified reference materials (LGC6188, NIST2768), which also served as the basis for calculating the standard uncertainty of the PAH measurements.

#### **S2. Quality assurance and control**

Rigorous quality control (QC) frameworks were implemented to verify the accuracy (trueness) and precision of all the analytical findings. To maintain the truthfulness of the data, several systematic measures were integrated into the workflow. These included daily conditioning of the analytical instrumentation to guarantee consistent retention times, minimal blank interference, and a sustained detector response. Furthermore, blank levels were continuously tracked, allowing for the correction of analytical signals whenever necessary. Daily calibration routines were also performed to ensure the stability of both the signal magnitude and temporal retention. To account for potential drift, a bracketing procedure was employed, where a calibration standard was analyzed every three to five samples, with subsequent corrections applied as needed. All the measurements were strictly conducted within the validated linear range of the established calibration curves. Additionally, matrix-certified reference materials (CRMs) were utilized to optimize extraction settings, evaluate recovery efficiency, detect potential analyte degradation or contamination, and assess matrix effects during the analytical run. Comprehensive recovery controls were maintained, encompassing the entire process from initial sample preparation to final measurement. Finally, a detailed estimation of measurement uncertainty was performed, the methodology of which is described in detail in this section.

**S3. Uncertainty estimation**

To determine the measurement uncertainty for each of these 16 compounds, we performed multiple analyses of two matrix-certified reference materials (CRMs): LGC6188 and NIST2768. The calculation framework incorporated two primary uncertainty contributors: the deviation between the measured and theoretical values (trueness, derived from recovery assays) and the consistency of the results (precision, derived from repeated measurements). Importantly, the use of matrix CRMs allowed for the calculation of specific recovery rates for every individual PAH. These reference materials were processed and analyzed via a protocol identical to that applied to the field samples, which enabled us to account for uncertainties stemming from both sample preparation and the calibration and measurement phases. We consider this approach the most robust method for evaluating trueness in this type of analytical work. Throughout five distinct measurement sessions, new aliquots of the LGC6188 material were prepared, ensuring that they underwent the entire sample preparation sequence. Owing to restricted availability, the NIST material was included in only two of these sessions. Within each session, the specific aliquot under study was analyzed five times.

From these data, both recovery and repeatability were determined for each PAH. Using error propagation—where the components represented trueness (recovery) and precision (repeatability)—the standard uncertainty for each specific compound was established, leading to the final calculation of the standard uncertainty for the total PAH content. The results for individual compounds showed standard uncertainties ranging from 5% to 17%, while the standard uncertainty for the total PAH concentration was determined to be 14%. Importantly, the propagation of error for the total content was calculated via absolute concentration units rather than percentages. The use of percentage-based uncertainty for individual compounds in the propagation formula would result in an artificial overestimation of the overall measurement uncertainty.

**References**

Mansouri, E., Yousefi, V., Ebrahimi, V., Eyvazi, S., Hejazi, M. S., Mahdavi, M., Mesbahi, A., & Tarhriz, V. (2020). Overview of ultraviolet-based methods used in polycyclic aromatic hydrocarbons analysis and measurement. Separation Science Plus, 3(2), 112–120. <https://doi.org/10.1002/sscp.201900077>.


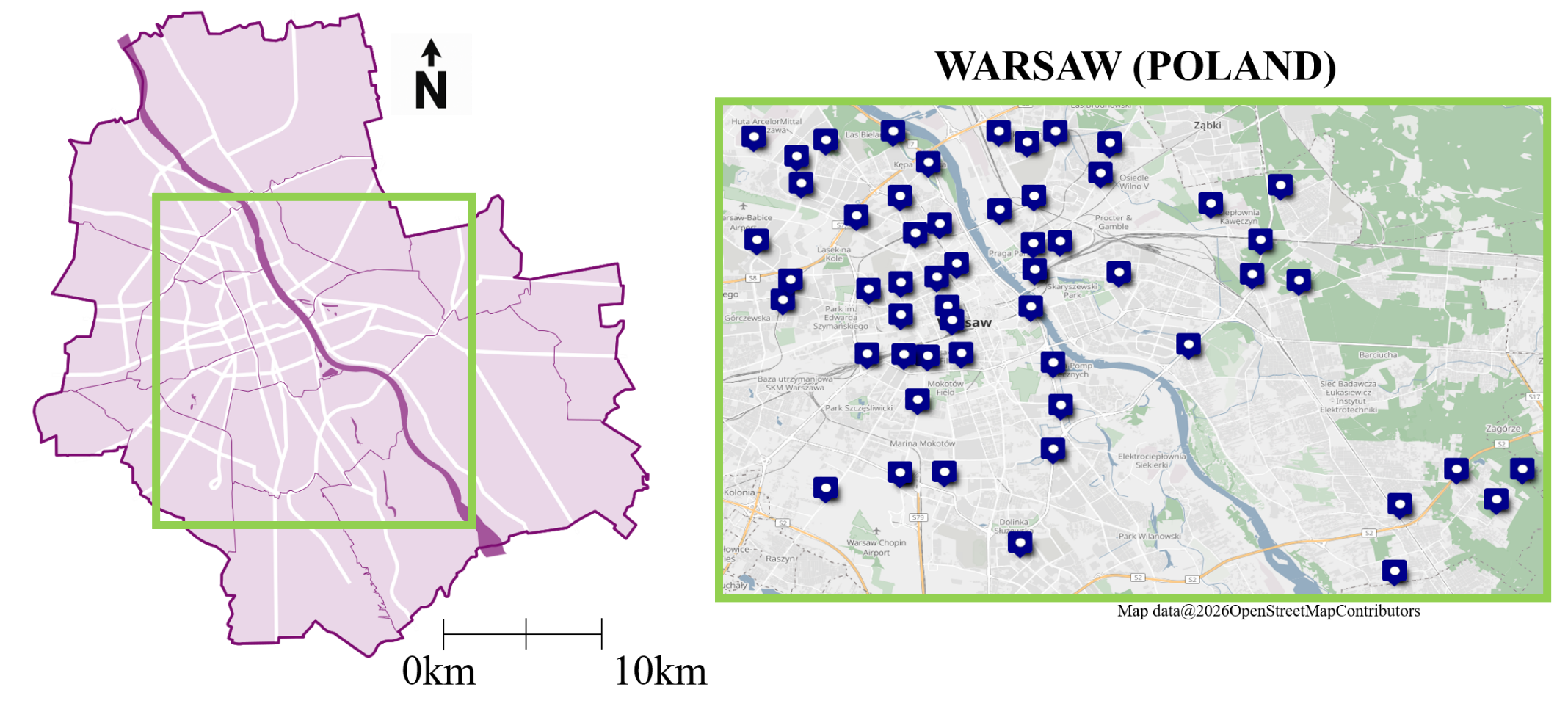


**Figure S1. S**ampling locations of street dust in Warsaw, Poland.

Figure S2. Mass percentage of the fraction of diameter <0.2 mm relative to the total sample mass.


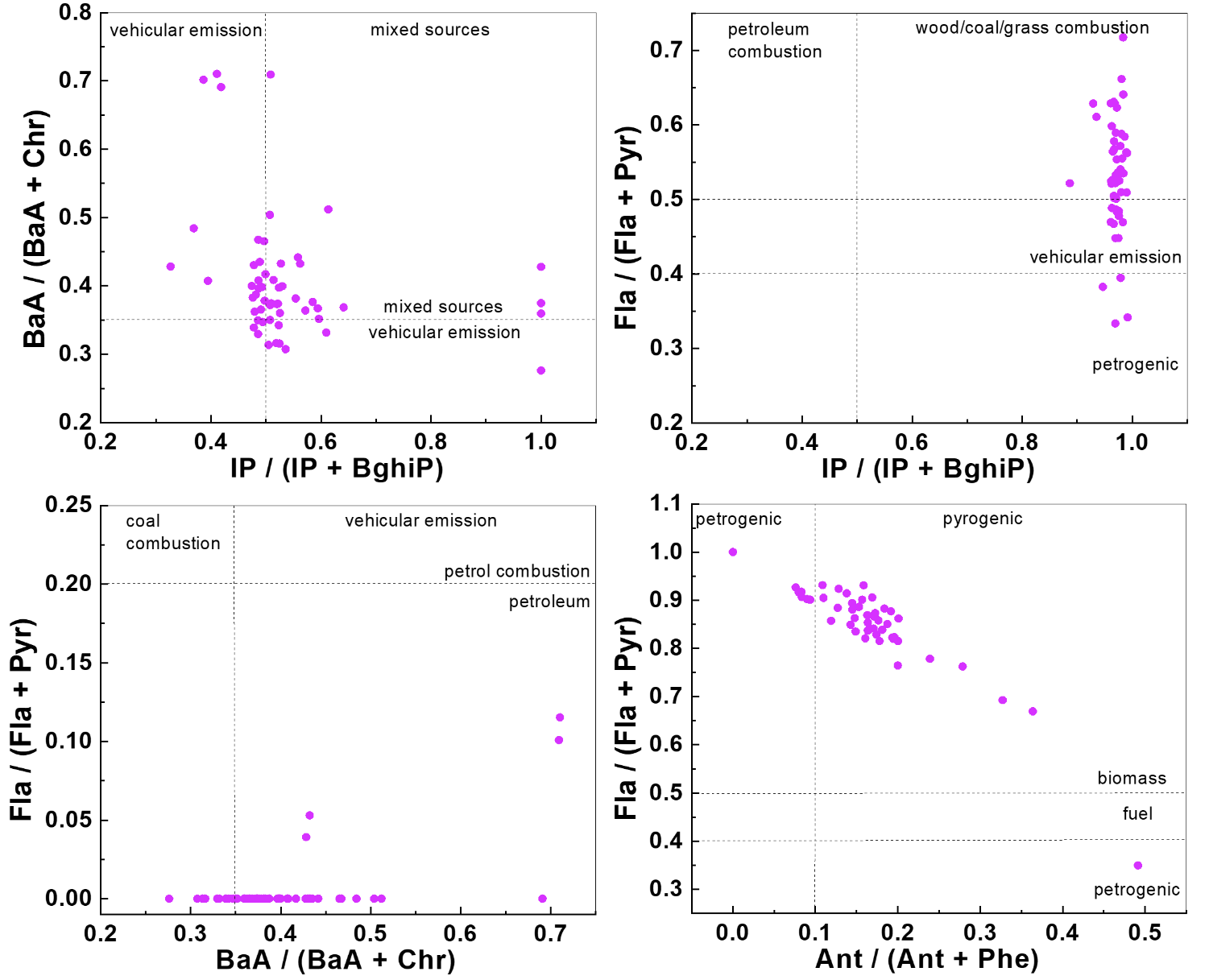


Figure S3. Isomeric ratios IP/(IP + BghiP) vs. BaA/(BaA + Chr), IP/(IP + BghiP) vs. Fla/(Fla + Pyr), BaA/(BaA + Chr) vs. Fla/(Fla + Pyr) and Ant/(Anr+Phe) vs. Fla/(Fla+Pyr).

Figure S4. Dendrogram of hierarchical cluster analysis.

Figure S5. Kernel density estimation (KDE) plot showing the relationship between standardized susceptibility scores and standardized total PAH concentrations and HMW PAH.

| **Basic separation and detection parameters** | |
| --- | --- |
| Instrument | HPLC Agilent 1290 with 1260 DAD |
| Elution | Gradient:  0.0 min 70% A  9.0 min 0% A  14.0 min 0% A  14.1 min 70% A  17.0 min 70% A |
| Flow rate | 0.35 ml/min |
| Injection volume | 5 µL |
| Column | Phenomenex Kinetex 3.5 µm PAH, 100*2.1 mm  @ 40^o^C |
| Detection | 254 nm, Ref = 400 nm |

Table S1. Chromatographic and detection parameters used in the determination of PAH.

| **Sample ID** | ** ** | **Traffic intensity**  **(veh./day)** | **Nap mg/kg** | **Acy mg/kg** | **Ace mg/kg** | **Flu mg/kg** | **Phe mg/kg** | **Ant mg/kg** | **Fla mg/kg** | **Pyr mg/kg** | **BaA mg/kg** | **Chr mg/kg** | **BbF mg/kg** | **BkF mg/kg** | **BaP mg/kg** | **DBA mg/kg** | **BghiP mg/kg** | **IP mg/kg** | ∑ **16 PAH mg/kg** |
| --- | --- | --- | --- | --- | --- | --- | --- | --- | --- | --- | --- | --- | --- | --- | --- | --- | --- | --- | --- |
|  | **10^-8^ m^3^/kg** |  |  |  |  |  |  |  |  |  |  |  |  |  |  |  |  |  |  |
|  | LOQ mg/kg |  | 0.118 | 0.0530 | 0.0300 | 0.0340 | 0.0280 | 0.0242 | 0.0430 | 0.0730 | 0.0190 | 0.0220 | 0.0110 | 0.00900 | 0.0150 | 0.00600 | 0.00900 | 0.0120 | 0.506 |
| 1 | 353 | 1900 | <LOQ | <LOQ | 0.591 | <LOQ | 0.168 | 0.0421 | 0.317 | 0.187 | 0.0420 | 0.110 | 0.0846 | 0.0467 | 0.129 | <LOQ | <LOQ | 0.113 | 2.79 |
| 2 | 570 | 12002 | <LOQ | <LOQ | 0.485 | <LOQ | 0.246 | 0.0833 | 0.403 | 0.395 | 0.117 | 0.237 | 0.148 | 0.0690 | 0.396 | <LOQ | 0.0692 | 0.108 | 3.12 |
| 3 | 395 | 12002 | <LOQ | <LOQ | 0.363 | <LOQ | 0.205 | 0.0596 | 0.425 | 0.251 | 0.0589 | 0.127 | 0.0849 | 0.0431 | 0.177 | <LOQ | 0.0730 | 0.0788 | 2.41 |
| 4 | 509 | 25626 | 0.196 | <LOQ | 1.027 | <LOQ | 0.379 | 0.0958 | 0.660 | 0.460 | 0.0787 | 0.177 | 0.132 | 0.0576 | 0.268 | <LOQ | 0.118 | 0.136 | 4.43 |
| 5 | 384 | 17500 | <LOQ | 1.51 | 0.940 | <LOQ | 0.150 | 0.0468 | 0.364 | 0.259 | 0.0774 | 0.117 | 0.0841 | 0.0478 | 0.178 | <LOQ | 0.0608 | 0.0591 | 4.19 |
| 6 | 387 | 40479 | <LOQ | 0.264 | 0.462 | <LOQ | 0.218 | 0.0500 | 0.302 | 0.233 | 0.0840 | 0.126 | 0.0972 | 0.0576 | 0.224 | <LOQ | 0.0902 | 0.102 | 2.78 |
| 7 | 422 | 9900 | <LOQ | <LOQ | 0.968 | <LOQ | 0.231 | 0.156 | 0.316 | 0.273 | 0.0893 | 0.145 | 0.107 | 0.0548 | 0.262 | <LOQ | 0.0723 | 0.0899 | 3.44 |
| 8 | 520 | 5023 | <LOQ | 1.05 | 0.539 | <LOQ | 0.153 | 0.0723 | 0.329 | 0.299 | 0.0626 | 0.127 | 0.0968 | 0.0507 | 0.221 | <LOQ | 0.112 | 0.106 | 3.53 |
| 9 | 440 | 28331 | 0.149 | <LOQ | 0.504 | <LOQ | 0.223 | 0.0791 | 0.366 | 0.329 | 0.0872 | 0.146 | 0.109 | 0.0573 | 0.253 | <LOQ | 0.0737 | 0.0799 | 2.75 |
| 10 | 399 | 14900 | <LOQ | <LOQ | 0.926 | <LOQ | <LOQ | 0.0513 | 0.323 | 0.296 | 0.0761 | 0.125 | 0.100 | 0.0711 | 0.223 | <LOQ | 0.0989 | 0.0980 | 3.05 |
| 11 | 378 | 14900 | <LOQ | 0.147 | 0.481 | <LOQ | 0.140 | 0.0667 | 0.214 | 0.172 | 0.0630 | 0.0955 | 0.0740 | 0.0426 | 0.180 | <LOQ | 0.0656 | 0.0721 | 2.48 |
| 12 | 336 | 39739 | <LOQ | <LOQ | <LOQ | <LOQ | 0.144 | <LOQ | 0.213 | 0.195 | 0.0756 | 0.0955 | 0.0727 | 0.0415 | 0.168 | <LOQ | 0.0437 | 0.0553 | 1.42 |
| 13 | 478 | 14900 | <LOQ | <LOQ | 1.00 | <LOQ | 0.524 | 0.0880 | 0.814 | 0.890 | 0.269 | 0.309 | 0.249 | 0.188 | 0.573 | 0.0837 | 0.141 | 0.139 | 5.59 |
| 14 | 672 | 14900 | <LOQ | 1.46 | 1.11 | 0.0392 | 0.798 | 0.187 | 1.24 | 0.962 | 0.442 | 0.590 | 0.497 | 0.287 | 1.03 | 0.121 | 0.213 | 0.103 | 9.51 |
| 15 | 531 | 18000 | <LOQ | 4.92 | 3.31 | <LOQ | 0.265 | 0.0448 | 0.564 | 0.544 | 0.160 | 0.277 | 0.204 | 0.130 | 0.420 | <LOQ | 0.127 | 0.122 | 11.99 |
| 16 | 695 | 41951 | <LOQ | <LOQ | 1.69 | <LOQ | 0.274 | 0.0362 | 0.345 | 0.293 | 0.105 | 0.159 | 0.131 | 0.0718 | 0.298 | <LOQ | 0.0882 | 0.0837 | 3.83 |
| 17 | 398 | 13617 | <LOQ | <LOQ | 0.416 | <LOQ | 0.344 | 0.0222 | 0.246 | 0.244 | 0.113 | 0.150 | 0.127 | 0.0836 | 0.252 | <LOQ | 0.0874 | 0.0801 | 2.37 |
| 18 | 410 | 41579 | <LOQ | <LOQ | 0.509 | <LOQ | 0.213 | 0.0369 | 0.500 | 0.302 | 0.086 | 0.144 | 0.109 | 0.0593 | 0.243 | <LOQ | 0.0852 | 0.0886 | 3.10 |
| 19 | 422 | 22249 | <LOQ | <LOQ | 0.318 | <LOQ | 0.106 | 0.0335 | 0.109 | 0.134 | 0.0327 | 0.0710 | 0.0421 | <LOQ | 0.111 | <LOQ | 0.0425 | 0.0469 | 1.49 |
| 20 | 505 | 46824 | <LOQ | <LOQ | 1.11 | <LOQ | 0.135 | 0.1030 | 0.232 | 0.212 | 0.0534 | 0.0991 | 0.0786 | 0.0587 | 0.155 | <LOQ | 0.0799 | 0.0824 | 2.70 |
| 21 | 378 | 9900 | <LOQ | 0.542 | 0.534 | <LOQ | 0.227 | 0.0359 | 0.216 | 0.266 | 0.0650 | 0.112 | 0.0814 | 0.0355 | 0.194 | <LOQ | 0.0561 | 0.0822 | 3.19 |
| 22 | 400 | 34009 | <LOQ | <LOQ | 0.697 | <LOQ | 0.125 | 0.0332 | 0.354 | 0.207 | 0.0641 | 0.101 | 0.0770 | 0.0432 | 0.172 | <LOQ | 0.0907 | 0.0843 | 2.43 |
| 23 | 440 | 25626 | <LOQ | <LOQ | 0.351 | <LOQ | 0.188 | 0.0522 | 0.230 | 0.241 | 0.0685 | 0.115 | 0.0874 | 0.0506 | 0.202 | <LOQ | 0.0871 | 0.0951 | 2.48 |
| 24 | 415 | 32612 | <LOQ | <LOQ | 0.597 | <LOQ | 0.203 | 0.0493 | 0.277 | 0.296 | 0.104 | 0.156 | 0.134 | 0.0724 | 0.325 | <LOQ | 0.100 | 0.0907 | 3.16 |
| 25 | 447 | 23780 | <LOQ | <LOQ | 1.19 | <LOQ | 0.184 | 0.0273 | 0.248 | 0.264 | 0.0957 | 0.124 | 0.100 | 0.0662 | 0.239 | <LOQ | 0.0798 | 0.0763 | 3.06 |
| 26 | 454 | 36844 | <LOQ | <LOQ | 1.09 | <LOQ | 0.283 | 0.0588 | 0.449 | 0.404 | 0.129 | 0.188 | 0.139 | 0.0802 | 0.296 | <LOQ | 0.241 | 0.157 | 4.29 |
| 27 | 339 | 63967 | <LOQ | <LOQ | 0.463 | <LOQ | 0.281 | <LOQ | 0.528 | 0.411 | 0.129 | 0.215 | 0.151 | <LOQ | <LOQ | <LOQ | <LOQ | 0.0206 | 2.20 |
| 28 | 621 | 9900 | <LOQ | <LOQ | 1.19 | <LOQ | 0.484 | 0.108 | 0.617 | 0.471 | 0.147 | 0.193 | 0.141 | 0.0870 | 0.326 | <LOQ | 0.122 | 0.136 | 4.02 |
| 29 | 399 | 47177 | <LOQ | <LOQ | 0.316 | <LOQ | 0.094 | <LOQ | 0.336 | 0.225 | 0.0539 | 0.101 | 0.087 | 0.0489 | 0.201 | <LOQ | 0.0920 | 0.0899 | 2.33 |
| 30 | 393 | 46280 | <LOQ | <LOQ | 1.92 | <LOQ | 0.047 | 0.179 | 0.0960 | 0.185 | 0.0694 | 0.124 | 0.106 | <LOQ | 0.219 | <LOQ | <LOQ | 0.0321 | 3.79 |
| 31 | 567 | 52292 | <LOQ | 0.711 | 1.75 | <LOQ | 0.467 | 0.125 | 0.641 | 0.638 | 0.133 | 0.234 | 0.175 | 0.0904 | 0.412 | <LOQ | 0.210 | 0.194 | 6.45 |
| 32 | 472 | 46280 | <LOQ | 0.366 | 1.64 | <LOQ | 0.400 | 0.0908 | 0.529 | 0.464 | 0.118 | 0.210 | 0.151 | 0.0847 | 0.348 | <LOQ | 0.155 | 0.1714 | 5.57 |
| 33 | 476 | 88910 | <LOQ | <LOQ | 1.66 | <LOQ | 0.427 | 0.116 | 0.606 | 0.583 | 0.113 | 0.218 | 0.146 | 0.0812 | 0.299 | <LOQ | 0.105 | 0.115 | 5.59 |
| 34 | 1068 | 34575 | <LOQ | <LOQ | 1.07 | <LOQ | 0.292 | 0.0596 | 0.273 | 0.312 | 0.0982 | 0.166 | 0.108 | 0.0610 | 0.264 | <LOQ | 0.103 | 0.106 | 3.04 |
| 35 | 453 | 49900 | <LOQ | <LOQ | 1.25 | <LOQ | 0.327 | 0.0803 | 0.406 | 0.459 | 0.110 | 0.203 | 0.137 | 0.0647 | 0.315 | <LOQ | 0.0458 | 0.0676 | 3.89 |
| 36 | 393 | 49900 | <LOQ | 0.196 | 0.910 | <LOQ | 0.177 | 0.0292 | 0.246 | 0.172 | 0.0630 | 0.110 | 0.074 | 0.0403 | 0.199 | <LOQ | 0.0510 | 0.0681 | 3.36 |
| 37 | 543 | 31630 | <LOQ | 0.669 | 2.07 | 0.0479 | 0.776 | 0.189 | 0.985 | 0.856 | 0.344 | 0.451 | 0.264 | 0.0956 | 0.535 | <LOQ | 0.100 | 0.128 | 7.86 |
| 38 | 363 | 59000 | <LOQ | <LOQ | 0.924 | <LOQ | 0.118 | 0.0417 | 0.269 | 0.201 | 0.0703 | 0.121 | 0.0793 | 0.0461 | 0.186 | <LOQ | 0.0291 | 0.0521 | 2.36 |
| 39 | 406 | 65553 | <LOQ | <LOQ | 1.23 | <LOQ | 0.304 | 0.0619 | 0.289 | 0.254 | 0.0693 | 0.129 | 0.0794 | 0.0444 | 0.188 | <LOQ | 0.0933 | 0.0880 | 3.21 |
| 40 | 628 | 66900 | <LOQ | <LOQ | 1.63 | <LOQ | 0.409 | 0.0818 | 1.10 | 0.434 | 0.135 | 0.218 | 0.140 | 0.0746 | 0.289 | <LOQ | 0.100 | 0.0909 | 5.36 |
| 41 | 467 | 49900 | <LOQ | <LOQ | 0.922 | <LOQ | 0.176 | 0.0388 | 0.471 | 0.264 | 0.0854 | 0.124 | 0.0915 | 0.0509 | 0.211 | <LOQ | 0.0526 | 0.0556 | 3.38 |
| 42 | 634 | 49900 | <LOQ | 0.353 | 1.22 | <LOQ | 0.205 | 0.0282 | 0.308 | 0.325 | 0.0927 | 0.154 | 0.110 | 0.0526 | 0.254 | <LOQ | 0.0810 | 0.114 | 3.77 |
| 43 | 356 | 7500 | <LOQ | <LOQ | 1.98 | <LOQ | 0.208 | 0.0515 | 0.379 | 0.304 | 0.0688 | 0.151 | 0.089 | 0.0410 | 0.175 | <LOQ | 0.0777 | 0.0793 | 4.22 |
| 44 | 372 | 79079 | <LOQ | <LOQ | 0.462 | <LOQ | 0.304 | 0.108 | 0.380 | 0.345 | 0.115 | 0.110 | 0.116 | 0.0634 | 0.288 | <LOQ | 0.0838 | 0.133 | 3.32 |
| 45 | 375 | 79079 | <LOQ | <LOQ | 0.393 | <LOQ | 0.177 | 0.0583 | 0.257 | 0.233 | 0.0787 | 0.110 | 0.0814 | 0.0421 | 0.192 | <LOQ | 0.0583 | 0.0581 | 2.41 |
| 46 | 425 | 14990 | <LOQ | <LOQ | 1.59 | <LOQ | 0.342 | 0.0732 | 0.703 | 0.360 | 0.139 | 0.202 | 0.153 | 0.0895 | 0.330 | <LOQ | 0.109 | 0.103 | 5.18 |
| 47 | 462 | 9900 | <LOQ | <LOQ | <LOQ | 0.0172 | 0.126 | <LOQ | 0.0952 | 0.153 | 0.103 | 0.0420 | 0.0458 | 0.0368 | 0.106 | <LOQ | 0.0444 | 0.0459 | 0.82 |
| 48 | 329 | 2515 | <LOQ | <LOQ | <LOQ | <LOQ | 0.149 | <LOQ | 0.109 | <LOQ | 0.0989 | 0.0420 | 0.0313 | 0.0280 | 0.0840 | <LOQ | 0.0390 | 0.0246 | 0.61 |
| 49 | 286 | 66897 | <LOQ | <LOQ | 0.110 | 0.0594 | 0.315 | <LOQ | 0.297 | 0.456 | 0.251 | 0.102 | 0.0674 | 0.0626 | 0.171 | <LOQ | 0.0619 | 0.0432 | 2.00 |
| 50 | 418 | 11259 | <LOQ | <LOQ | <LOQ | <LOQ | 0.139 | 0.0494 | 0.450 | 0.266 | 0.114 | 0.121 | 0.1073 | 0.0780 | 0.208 | <LOQ | 0.2522 | 0.148 | 1.93 |
| 51 | 381 | 9900 | <LOQ | <LOQ | 1.40 | <LOQ | 0.126 | <LOQ | 0.374 | 0.273 | 0.074 | 0.100 | 0.0835 | 0.0488 | 0.168 | <LOQ | <LOQ | 0.0937 | 2.74 |
| 52 | 145 | 4900 | <LOQ | <LOQ | <LOQ | <LOQ | 0.134 | 0.0611 | 0.381 | 0.243 | 0.104 | 0.204 | 0.106 | 0.0717 | 0.205 | <LOQ | 0.134 | 0.123 | 1.77 |
| 53 | 239 | 7500 | <LOQ | <LOQ | <LOQ | <LOQ | 0.221 | <LOQ | 0.174 | 0.348 | 0.174 | 0.0778 | 0.070 | 0.0546 | 0.157 | 0.0122 | 0.0613 | 0.0442 | 1.39 |
| 54 | 251 | 13980 | <LOQ | <LOQ | <LOQ | <LOQ | 0.366 | 0.106 | 0.561 | 0.514 | 0.200 | 0.228 | 0.201 | 0.147 | 0.404 | <LOQ | 0.501 | 0.4740 | 3.70 |
| 55 | 164 | 1990 | <LOQ | <LOQ | <LOQ | <LOQ | <LOQ | <LOQ | 0.0931 | <LOQ | <LOQ | <LOQ | <LOQ | <LOQ | <LOQ | <LOQ | <LOQ | <LOQ | 0.467 |
| 56 | 113 | 13980 | <LOQ | <LOQ | 1.15 | <LOQ | 0.844 | 0.163 | 1.58 | 1.786 | 0.676 | 0.666 | 0.584 | 0.45 | 1.19 | <LOQ | 0.388 | 0.400 | 9.88 |
| 57 | 185 | 7500 | <LOQ | <LOQ | <LOQ | <LOQ | <LOQ | <LOQ | <LOQ | <LOQ | <LOQ | <LOQ | <LOQ | <LOQ | <LOQ | <LOQ | <LOQ | <LOQ | <LOQ |
| **Min.** | **113** | **1900** | **<LOQ** | **<LOQ** | **<LOQ** | **<LOQ** | **<LOQ** | **<LOQ** | **<LOQ** | **<LOQ** | **<LOQ** | **<LOQ** | **<LOQ** | **<LOQ** | **<LOQ** | **<LOQ** | **<LOQ** | **<LOQ** | **<LOQ** |
| **Max.** | **1068** | **88910** | **0.196** | **4.92** | **3.31** | **0.0594** | **0.844** | **0.243** | **1.58** | **1.79** | **0.676** | **0.666** | **0.5839** | **0.453** | **1.19** | **0.121** | **0.501** | **0.4740** | **12.0** |
| **Mean** | **429** | **30564** | **0.0639** | **0.237** | **0.850** | **0.00347** | **0.256** | **0.0701** | **0.414** | **0.354** | **0.118** | **0.164** | **0.123** | **0.0704** | **0.265** | **0.0039** | **0.0974** | **0.101** | **3.56** |
| **SD** | **146** | **22359** | **0.0211** | **0.703** | **0.663** | **0.0115** | **0.173** | **0.0573** | **0.282** | **0.267** | **0.104** | **0.114** | **0.0935** | **0.0675** | **0.195** | **0.0191** | **0.0858** | **0.0751** | **2.17** |

Table S2. Concentrations of 16 priority PAHs, magnetic susceptibility, and the total PAH content (Σ16PAHs) in the analyzed road dust samples.

|  | **ERMEffects range-median [mg]kg]** |
| --- | --- |
| **Nap*** | 2.10 |
| **Acy*** | 0.640 |
| **Ace*** | 0.500 |
| **Flu*** | 0.540 |
| **Phe*** | 1.50 |
| **Ant*** | 1.10 |
| **Fla*** | 5.10 |
| **Pyr*** | 2.60 |
| **BaA*** | 1.60 |
| **Chr*** | 2.80 |
| **BbF***** | 1.80 |
| **BkF**^and^***** | 1.62 |
| **BaP*** | 1.60 |
| **DBA**^and^***** | 0.260 |
| **BghiP*** | 9.60 |
| **IP*** | 9.60 |

***Long et al., 1995** **<https://doi.org/10.1007/BF02472006>), **Han et al., 2012 ([10.1016/j.marpolbul.2020.111800](https://doi.org/10.1016/j.marpolbul.2020.111800)); ***Ramzi et al. (2017) (https://doi.org/10.1016/j.marpolbul.2016.10.015).**

Table S3**.** Selected sediment quality guidelines (mg/kg dry mass) of PAH for ecological risk assessments.

| **Sample ID** | ∑LMW mg/kg | ∑HMW mg/kg | LMW/HMW |
| --- | --- | --- | --- |
| 1 | 0.903 | 1.03 | 0.875 |
| 2 | 0.917 | 1.95 | 0.471 |
| 3 | 0.731 | 1.32 | 0.553 |
| 4 | 1.75 | 2.09 | 0.834 |
| 5 | 2.72 | 1.25 | 2.17 |
| 6 | 1.07 | 1.32 | 0.811 |
| 7 | 1.46 | 1.41 | 1.03 |
| 8 | 1.89 | 1.41 | 1.34 |
| 9 | 0.998 | 1.51 | 0.663 |
| 10 | 1.09 | 1.41 | 0.773 |
| 11 | 0.910 | 0.98 | 0.927 |
| 12 | 0.275 | 0.96 | 0.285 |
| 13 | 1.72 | 3.66 | 0.470 |
| 14 | 3.66 | 5.48 | 0.668 |
| 15 | 8.61 | 2.55 | 3.38 |
| 16 | 2.11 | 1.58 | 1.34 |
| 17 | 0.888 | 1.39 | 0.640 |
| 18 | 0.864 | 1.62 | 0.534 |
| 19 | 0.564 | 0.592 | 0.952 |
| 20 | 1.45 | 1.05 | 1.38 |
| 21 | 1.42 | 1.11 | 1.27 |
| 22 | 0.931 | 1.20 | 0.777 |
| 23 | 0.667 | 1.18 | 0.566 |
| 24 | 0.925 | 1.56 | 0.593 |
| 25 | 1.48 | 1.30 | 1.14 |
| 26 | 1.50 | 2.09 | 0.721 |
| 27 | 0.820 | 1.46 | 0.562 |
| 28 | 1.86 | 2.24 | 0.829 |
| 29 | 0.486 | 1.24 | 0.393 |
| 30 | 2.22 | 0.834 | 2.66 |
| 31 | 3.13 | 2.73 | 1.15 |
| 32 | 2.57 | 2.23 | 1.15 |
| 33 | 2.31 | 2.27 | 1.02 |
| 34 | 1.52 | 1.49 | 1.02 |
| 35 | 1.76 | 1.81 | 0.972 |
| 36 | 1.39 | 1.03 | 1.35 |
| 37 | 3.78 | 3.76 | 1.00 |
| 38 | 1.19 | 1.06 | 1.13 |
| 39 | 1.70 | 1.24 | 1.38 |
| 40 | 2.23 | 2.59 | 0.860 |
| 41 | 1.24 | 1.41 | 0.882 |
| 42 | 1.56 | 1.49 | 1.04 |
| 43 | 2.34 | 1.37 | 1.71 |
| 44 | 0.980 | 1.64 | 0.598 |
| 45 | 0.734 | 1.11 | 0.659 |
| 46 | 2.11 | 2.19 | 0.965 |
| 47 | 0.247 | 0.675 | 0.365 |
| 48 | 0.255 | 0.460 | 0.553 |
| 49 | 0.573 | 1.52 | 0.378 |
| 50 | 0.309 | 1.75 | 0.177 |
| 51 | 1.65 | 1.22 | 1.36 |
| 52 | 0.315 | 1.58 | 0.200 |
| 53 | 0.366 | 1.17 | 0.312 |
| 54 | 0.593 | 3.23 | 0.183 |
| 55 | 0.156 | 0.178 | 0.875 |
| 56 | 2.26 | 7.73 | 0.293 |
| 57 | 0.160 | 0.112 | 1.43 |
| **Minimum** | 0.156 | 0.112 | 0.177 |
| **Maximum** | 8.61 | 7.73 | 3.38 |
| **Mean** | 1.48 | 1.72 | 0.923 |
| **SD** | 1.27 | 1.19 | 0.568 |

Table S4. Sum of low molecular weight (∑LMW) and high molecular weight (∑HMW) PAHs, and the LMW/HMW ratio in road dust samples.

Table S5. Distribution of Self-Organizing Map (SOM) weights.
